# Genetic and pharmaceutical manipulation of H3K9 methyltransferase Suv39h1 promotes liver regeneration by unleashing HMGB2 transcription

**DOI:** 10.1038/s12276-026-01677-4

**Published:** 2026-04-10

**Authors:** Yunjie Lu, Jiawen Zhou, Xiulian Miao, Sheng Zeng, Lei Qin, Yong Xu, Shuai Wang, Zilong Li

**Affiliations:** 1 Department of General Surgery, the First Affiliated Hospital of Soochow University, Suzhou, China; 2 State Key Laboratory of Natural Medicines, Department of Pharmacology, China Pharmaceutical University, Nanjing, China; 3 Institute of Biomedical Research, College of Agriculture and Life Sciences, Liaocheng University, Liaocheng, China; 4 Stem Cell Center, Affiliated Drum Tower Hospital of Nanjing University Medical School, Nanjing, China; 5 Division of Hepatobiliary and Transplantation Surgery, Department of General Surgery, Nanjing Drum Tower Hospital, the Affiliated Hospital of Nanjing University Medical School, Nanjing, China; 6 Department of Endocrinology, Jinan Central Hospital Affiliated to Shandong First Medical University, Jinan, China; 7 Institute of Brain Science and Brain-Inspired Research, Shandong First Medical University and Shandong Academy of Medical Sciences, Jinan, China

**Keywords:** Liver diseases, Hepatocytes

## Abstract

Robust liver regeneration counteracts and facilitates recovery from liver injuries. The underlying epigenetic mechanisms, however, are not fully understood. Here we investigated the role of suppressor of variegation 3-9 homolog 1 (Suv39h1), a histone H3K9 methyltransferase, in liver regeneration. Suv39h1 expression was repressed by DNMT1 during liver regeneration. Systemic or hepatocyte-specific deletion of Suv39h1 in mice enhanced liver regeneration and post-surgery survival following partial hepatectomy. RNA sequencing revealed high-mobility group protein B2 (HMGB2) as a target for Suv39h1. Suv39h1 downregulation in proliferating hepatocytes allowed E2F1 to activate HMGB2 transcription. Consistently, HMGB2 knockdown attenuated proliferation of hepatocytes in response to HGF treatment and suppressed liver regeneration in mice. Integrated transcriptomic analysis indicated that HMGB2 may contribute to proliferation of hepatocytes by regulating a panel of proregenerative genes. Importantly, Suv39h1 inhibition by chaetocin boosted liver regeneration in mice. Finally, a significant correlation between Suv39h1, HMGB2 and proliferative markers was identified in patients with acute liver failure. In conclusion, our data uncover an unrecognized role for Suv39h1 in liver regeneration. Therefore, targeting Suv39h1 may be considered as a viable strategy to boost liver regeneration after injury.

## Suv39h1 inhibition enhances liver regeneration after injury

The liver can regenerate itself after damage but this process can be disrupted, leading to liver failure. Researchers focused on a protein called Suv39h1, which affects liver regeneration. They used mice with and without the *Suv39h1* gene. They performed surgeries to remove parts of the liver and observed how well the liver regenerated. They found that mice lacking *Suv39h1* had accelerated liver regeneration. This was due to increased expression of genes, *HMGB2* in particular, that promoted cell growth. By deleting/inhibiting *Suv39h1*, *HMGB2* expression was increased, aiding liver regeneration. Researchers concluded that targeting *Suv39h1* could be a new way to enhance liver healing. In the future, this approach might lead to treatments for chronic liver diseases by boosting the body’s natural ability to repair itself.

This summary was initially drafted using artificial intelligence, then revised and fact-checked by the author.

## Introduction

Exposure to toxins, medications, corrosive chemicals, ischemia or trauma can lead to extensive death of hepatocytes and consequently massive loss of the liver parenchyma. By re-entering the cell cycle, dormant hepatocytes can resume proliferation to compensate for the loss of liver parenchyma^1^. Robust liver regeneration following injury may be sufficient to overcome the loss of liver mass and restore normal liver function, thus protecting against liver failure. Compromised liver regeneration, however, may render an individual susceptible to liver insufficiency and increase liver failure-associated mortality^2^. Therefore, identification of core regulatory molecules and the governing mechanisms that contribute to liver regeneration holds the key to the development of novel therapeutic solutions to treat liver injury^3^.

Following liver injury, the remnant liver parenchyma is exposed to a sea of cytokines and growth factors that collectively modulate the regenerative response. Hepatocyte growth factor (HGF) is among the best characterized pro-proliferative factors contributing to liver regeneration^4^. HGF levels are up-regulated following liver injury induced by partial hepatectomy (PHx) or exposure to hepatotoxic substances paralleling active proliferation of hepatocytes^5^. Importantly, serum HGF levels are found to be elevated in patients receiving orthotopic liver transplantation and appear to predict post-transplant liver function^6,7^. Later studies have found that HGF is primarily derived from liver sinusoidal endothelial cells^8^. Exposure of primary hepatocytes to HGF provokes DNA synthesis more potently than other pro-proliferative growth factors such as EGF, with no effect on nonparenchymal liver cells^9^. HGF exerts its pro-proliferative effects via binding to the transmembrane receptor c-Met, as evident from the observation that mice harboring c-Met deletion display impaired liver regeneration following PHx^10^. The HGF–c-Met axis promotes proliferation of hepatocytes through a complex network of signaling cascades with characteristic changes in cellular transcriptome, of which the underlying regulatory mechanism is not completely understood.

The regenerative potential of the liver is underscored by dynamic alterations of chromatin status that skew cell fate determination, in which the epigenetic machinery plays a major role. Post-translational modifications of histone tails constitute a major branch of the mammalian epigenetic machinery. Mounting evidence alludes to a correlation between differential histone modifications and liver regeneration. For instance, Shi et al. have observed a global elevation of histone lysine acetylation in the murine liver following PHx that presumably contributes to hepatocyte proliferation^11^. Sato et al. have noted an up-regulation of trimethylated histone H3 lysine 4 (H3K4), typically associated with transcriptional activation, and a simultaneous downregulation of trimethyl H3 lysine 9 (H3K9), usually detected at the silenced chromatin region, in the (regenerating) livers of mice subjected to PHx^12^. More recently, the Sadler group profiled chromatin states embedded in quiescent and proliferating hepatocytes that reprogram lineage-specific transcription and contribute to liver regeneration^13^. Using a combination of highly sophisticated transcriptomic techniques that include single-cell RNA sequencing (scRNA-seq), chromatin immunoprecipitation followed by sequencing (ChIP-seq), assay for transposase-accessible chromatin using sequencing (ATAC-seq) and enhanced reduced representation bisulfite sequencing (ERRBS-seq), these authors propose that the genes in hepatocytes can be categorized into six groups, each barcoded with a distinct set of histone and DNA modifications, whereas erasure of trimethyl H3K27 appears to be a rate-limiting event that catapults quiescent hepatocytes into active cell cycling. H3K9 trimethylation in mammals is exclusively catalyzed by SUV39H^14^. Two SUV39H paralogues, Suv39h1 and Suv39h2, have been identified; Suv39h1 is ubiquitously expressed, whereas Suv39h2 expression may exhibit certain tissue preference^15^. SUV39H has a well-established role in demarcating and maintaining the silenced state of the heterochromatin region, as Suv39h1/Suv39h2 double-knockout mice die prematurely owing to widespread genomic instability^16^. However, little is known whether SUV39H-mediated transcription in the euchromatin region is linked to human diseases. Recently, it has been suggested that aberrant activation of SUV39H aggravates the development and progression of ischemic heart disease^17,18^ and nonalcoholic steatohepatitis^19^. Here, we report that systemic deletion or hepatocyte-specific ablation of Suv39h1 accelerates liver regeneration in mice. Therefore, targeting Suv39h1 may unlock the door toward the development of novel therapeutics to boost liver regeneration.

## Methods

### Animals

All animal experiments were reviewed and approved by the intramural Committee on Ethical Treatment of Experimental Animals. Suv39h1-knockout (KO), Suv39h2-KO and wild-type (WT) littermate hepatocyte-specific deletions of Suv39h1 were achieved by crossing the *Suv39h1*^f/f^ strain with the *Alb*-Cre strain. C57BL/6 mice were purchased from GemPharmatech. Global Suv39h1-KO mice (*Suv39h1*^−/−^), in which exon 2 and exon 3 were deleted by homologous recombination, and global Suv39h2-KO (*Suv39h2*^−/−^) mice, in which exon 2 was deleted by homologous recombination, have been described previously^17^. To generate hepatocyte-specific Suv39h1-KO mice, *Suv39h1*^f/f^ mice^20^, in which exons 2 through 3 were floxed, were crossbred with *Alb*-Cre mice^21^. *Suv39h1*^+/-^ (*Suv39h2*^+/-^) mice were crossed to generate *Suv39h1*^−/−^ (*Suv39h1*^−/−^) mice whereas the *Suv39h1*^+/+^ (*Suv39h2*^+/+^) mice in the same litter were used as WT control.

To investigate liver regeneration, two animal models were exploited. In the first model, PHx was performed in 6-week-old male mice as previously described^22,23^. For 2/3 PHx, the mice were anesthetized with 2% isoflurane (RWD Life Sciences, R510-22-10) and a midline incision was created to expose the xiphoid process. We placed a silk thread on the base of the left lateral lobe, tied the two ends of the suture over the top of the left lateral lobe and removed the tied lobe just above the suture with a microsurgical scissor. Thereafter, we placed a thread for the second knot between the stump and the median lobe and removed the tied median lobe above the suture. For 4/5 PHx, the caudate lobe, in addition to the median and left lateral lobe, was surgically removed. After the surgery, the mice were placed on a heating pad for recovery before being transferred back to the cage. In the second model, 8-week-old male mice were injected peritoneally with a single dose of CCl_4_ (25% solution dissolved in corn oil, 1 μl/g) and killed 48 h after the injection as previously described^24–26^. In certain experiments, chaetocin (Selleck, S8068) was administered daily via intraperitoneal injection for three consecutive days (0.25 mg/kg) before the PHx surgery or CCl_4_ injection. In certain experiments, Hmgb2-targeting short hairpin RNA (shRNA) (CAGCUAAACUAAAGGAGAATT) was placed downstream of the human thyroxin binding globulin (TBG) promoter, packed into AAV8 and injected into the mice via the tail vein (1 × 10^11^ GC/mouse) 2 weeks before the PHx procedure.

### Histology

Histological analyses were performed essentially as described before. Paraffin sections were blocked with 10% normal goat serum for 1 h at room temperature and then incubated with anti-Ki67 (Abcam, ab16667, 1:200). Staining was visualized by incubation with anti-rabbit secondary antibody and developed with a streptavidin-horseradish peroxidase kit (Pierce, 36000) for 20 min. Pictures were taken using an Olympus IX-70 microscope. For each mouse, at least three slides were stained and at least five different fields were analyzed for each slide. Slides were observed under a light microscope at high power (×40) by two pathologists independently in a double-blind fashion. The number of Ki67+ cells and total number of cells in each slide were counted and divided. Data are expressed as relative Ki67 staining as a percentage of total cells.

### Cell culture, plasmids and transient transfection

Primary mouse hepatocytes were isolated and maintained as previously described^27^. In brief, the mice were anesthetized with ketamine (112.5 mg/kg) and xylazine (22.5 mg/kg). The livers were digested with collagenase IV (Thermo Fisher, 17104019) and passed through a 70-μm cell strainer (Corning, 431751). The cell suspensions were mixed with Percoll (Sigma, P4937) and centrifuged at 200*g* for 10 min at 4 °C. The yielded hepatocytes were resuspended and plated in culture dishes with DMEM (Thermo Fisher, 11965092) supplemented with fetal bovine serum (Thermo Fisher, A5670901). Cell viability was examined at the time of seeding by trypan blue staining; typical isolation yielded >95% viability.

The *Hmgb2* promoter–luciferase construct was generated by amplifying genomic DNA spanning the proximal promoter and the first exon of the *Hmgb2* gene (−1000/+100) and ligating into a pGL4-basic vector (Promega, E6651). The QuickChange II kit (Agilent, 200519) was used for mutagenesis. Transient transfections were performed with Lipofectamine LTX (Thermo Fisher, 15338100). The cells were seeded 16–24 h before transfection at a density of 1 × 10^5^ cells/well in 12-well tissue culture dishes. An enhanced green fluorescent protein expression construct was included in each well to monitor transfection efficiency. Luciferase activities were assayed at 24–48 h after transfection using a luciferase reporter assay system (Promega, E1500) as previously described^28^. Recombinant murine HGF (R&D, 2207-HG) was reconstituted in sterile PBS and added to the hepatocytes at a final concentration of 20 ng/ml. The cells were treated with HGF for 24 h before collection.

### Protein extraction and Western blotting

Tissue and cell lysates were obtained by resuspending pellets in RIPA buffer (50 mM Tris pH7.4, 150 mM NaCl, 1% Triton X-100), with freshly added protease inhibitors (Thermo Fisher, A32955) as previously described^29^. Equal amounts of proteins (~25 μg) were separated by 10% mini SDS–polyacrylamide gel electrophoresis gels and transferred to nitrocellulose membranes (Bio-Rad, 1620112). The membranes were blocked with 5% milk powder in TBST buffer at room temperature for 1 h and then hybridized at room temperature to the following commercially available antibodies: anti-cyclin B1 (Proteintech, 28603-1), anti-cyclin D1 (Proteintech, 26939-1), anti-c-Myc (Proteintech, 10828-1), anti-HMGB2 (Proteintech, 14579-1), anti-Suv39h1 (Genetex, GTX112263) and anti-β-actin (Sigma, A2228) antibodies. For densitometric quantification, densities of target proteins were normalized to those of β-actin. Data are expressed as relative protein levels compared with the control group, which is arbitrarily set as 1.

### Statistical analysis

Data are presented as mean ± s.d. For experiments concerning multiple groups, one-way analysis of variance (ANOVA) with post hoc Scheffe’s analyses were performed to evaluate the differences. The differences between two (control and experimental) groups were determined using a two-sided, unpaired Student’s *t*-test. *P* values smaller than 0.05 are considered significant.

## Results

### Suv39h1 was repressed by DNMT1-mediated DNA methylation in hepatocytes

It was observed that Suv39h1 levels were markedly but transiently down-regulated in the liver following PHx (2/3 PHx), whereas no change in Suv39h2 expression was detected (Supplementary Fig. 1a, b). It was also interesting to note that DNA methyltransferase 1 (DNMT1), but not DNMT3a/3b, was up-regulated at the same time mirroring the alteration of Suv39h1 (Supplementary Fig. 1c). ChIP assay showed that DNMT1 was recruited to the CpG island located on the *Suv39h1* promoter leading to accumulation of 5-methylcytosine (Supplementary Fig. 1d). In primary hepatocytes, DNMT1 knockdown erased CpG methylation and normalized Suv39h1 expression (Supplementary Fig. 2). Together, these data suggest that DNMT1-dependent DNA methylation may dynamically regulate Suv39h1 expression during liver regeneration.

#### Suv39h1 deficiency promotes liver regeneration in mice

**Fig. 1:**
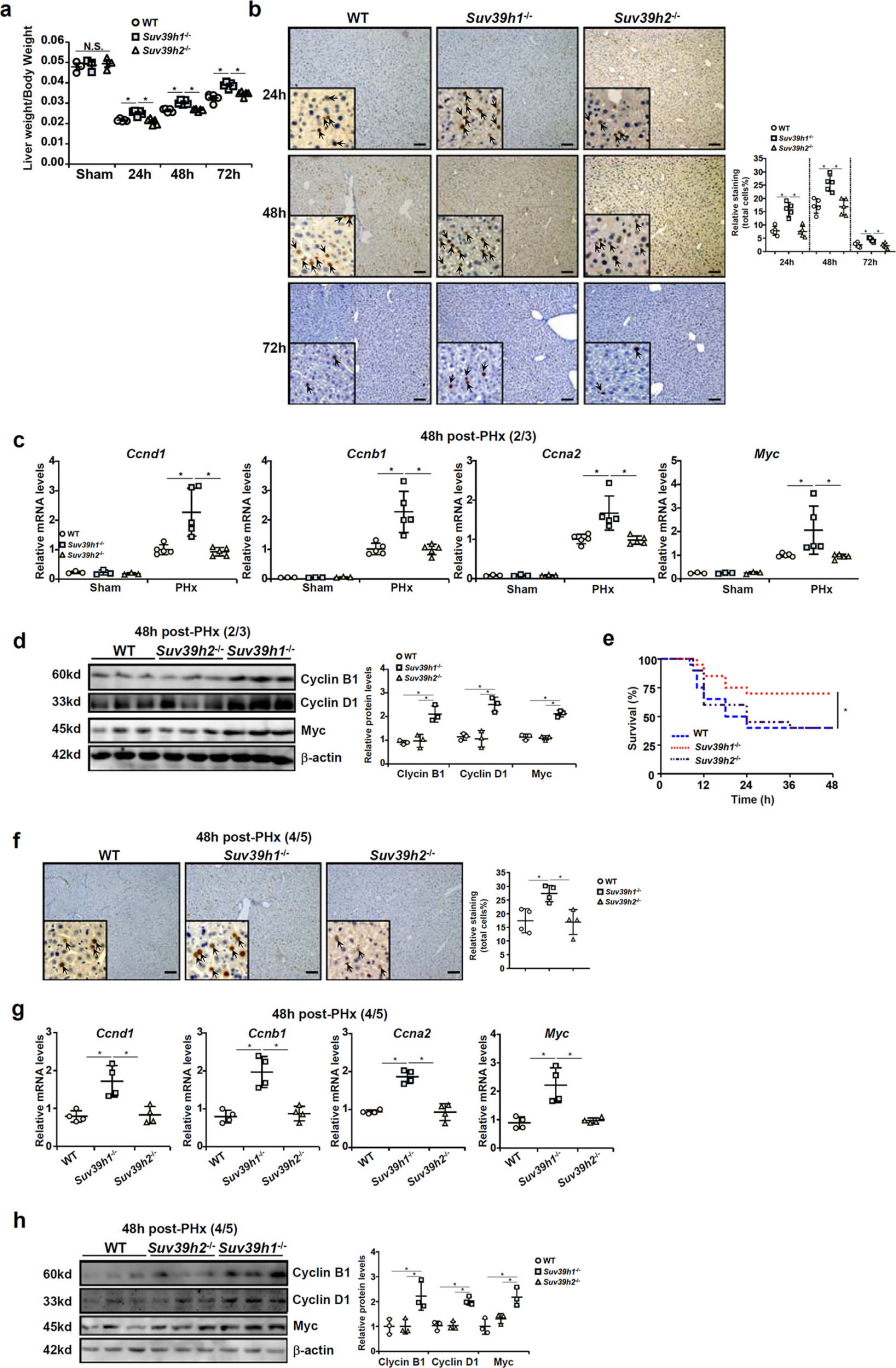
Suv39h1 deficiency promotes liver regeneration in mice. **a**–**d** Suv39h1^−/−^ mice, Suv39h2^−/−^ mice and WT mice were subjected to 2/3 PHx and killed at indicated time points. The image shows liver weight versus body weight (**a**). Paraffin sections were stained for Ki67 (**b**) and gene expression levels were examined by qPCR (**c**) and Western blotting (**d**). *N* = 3 mice for the sham groups and *N* = 5 mice for the PHx groups. **e**–**h** Suv39h1^−/−^ mice, Suv39h2^−/−^ mice and WT mice were subjected to 4/5 PHx and monitored for 48 h after surgery. The image shows the Kaplan–Meier plot (**e**). *N* = 20 mice for each group. Paraffin sections were stained for Ki67. Quantifications were performed with Image Pro (**f**) and gene expression levels were examined by qPCR (**g**) and Western blotting (**h**). *N* = 5 mice for each group. Data are expressed as mean ± s.d. ^*^P < 0.05, one-way ANOVA with post hoc Scheffe’s analyses.

Systemic deletion of Suv39h1 or Suv39h2 (see Supplementary Fig. 3 for validation) did not alter gross hepatic anatomy or liver weight under physiological conditions (data not shown). Nor did Suv39h1/h2 deletion impact liver weight/body weight following the sham procedure (Fig. 1a). Suv39h1 deficiency, but not Suv39h2 deficiency, significantly accelerated liver regeneration following 2/3 PHx as measured by liver weight/body weight at 1 day, 2 days and 3 days after the surgical procedure (Fig. 1a). Enhanced liver regeneration by Suv39h1 deletion might be accounted for by, at least in part, augmented proliferation of hepatocytes as assessed by Ki67 staining (Fig. 1b). Quantitative PCR (Fig. 1c) and Western blotting (Fig. 1d) revealed that Suv39h1 loss of function caused up-regulation of pro-proliferative genes. We then performed 4/5 PHx in the mice to determine whether Suv39h1 deficiency might improve post-surgery survival. As shown in Fig. 1e, Suv39h1-KO mice, but not Suv39h2-KO mice, exhibited a significant advantage over their WT littermates in terms of surviving lethal hepatectomy. Examination of those mice that survived the surgery indicated that hepatocyte proliferation was stronger, whereas levels of proregenerative genes were significantly higher, in Suv39h1-KO mice than in either the Suv39h2-KO mice or the WT mice (Fig. 1f–h). In a second model of liver regeneration in which the mice were injected with a single dose of carbon tetrachloride (CCl_4_), Suv39h1 deletion alleviated liver injury, as measured by plasma ALT levels and AST levels, and enhanced liver regeneration (Supplementary Fig. 4).

#### Hepatocyte-specific Suv39h1 deletion promotes liver regeneration in mice

We next asked whether hepatocyte-conditional Suv39h1-KO mice (*Suv39h1*^f/f^; *Alb*-Cre, LKO) would phenocopy the systemic Suv39h1-KO mice when subjected to PHx (see Supplementary Fig. 5 for LKO efficiency and specificity). Primary hepatocytes isolated from the LKO mice displayed stronger potency in proliferation, compared with those from the WT mice, when exposed to HGF treatment, as determined by the expression of proregenerative genes and by EdU incorporation assay (Supplementary Fig. 6).

**Fig. 2:**
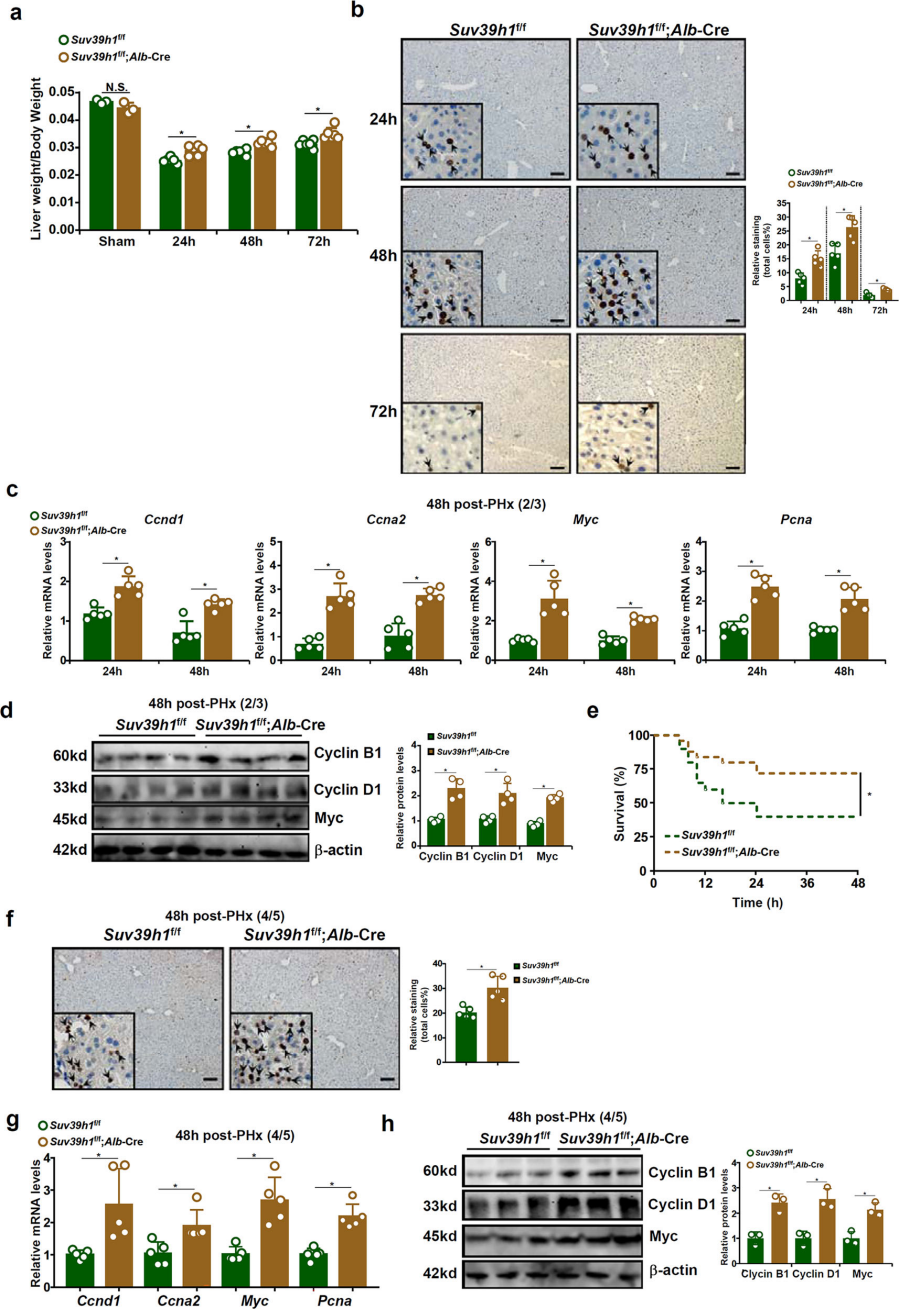
Hepatocyte-specific Suv39h1 deletion promotes liver regeneration in mice. **a**–**d** Suv39h1^LKO^ mice and WT mice were subjected to 2/3 PHx and killed at indicated time points. The image shows liver weight versus body weight (**a**). Paraffin sections were stained for Ki67. Quantifications were performed with Image Pro (**b**) and gene expression levels were examined by qPCR (**c**) and Western blotting (**d**). *N* = 3 mice for the sham groups and *N* = 5 mice for the PHx groups. **e**–**h** Suv39h1^LKO^ mice and WT mice were subjected to 4/5 PHx and monitored for 48 h after surgery. The image shows the Kaplan–Meier plot (**e**). *N* = 20 mice for each group. Paraffin sections were stained for Ki67. Quantifications were performed with Image Pro (**f**) and gene expression levels were examined by qPCR (**g**) and Western blotting (**h**). *N* = 5 mice for each group. Data are expressed as mean ± s.d. ^*^P < 0.05, two-tailed Student’s *t*-test.

When both the LKO mice and the WT mice were subject to 2/3 hepatectomy, the LKO mice regained considerable more liver weight compared with the control mice at 1 day, 2 days and 3 days after the surgery, although there was no difference between these mice in terms of liver weight/body weight under physiological conditions (Fig. 2a). Ki67 staining revealed that proliferation of hepatocytes was more robust in the LKO mice than in control mice (Fig. 2b). Furthermore, hepatocyte-specific Suv39h1 deletion was sufficient to cause an up-regulation of pro-proliferative genes as measured by quantitative PCR (qPCR) (Fig. 2c) and Western blotting (Fig. 2d). Again, in the lethal PHx model, the LKO mice displayed significantly improved survival compared with the control mice when monitored over 48 h after surgery (Fig. 2e). Consistently, proliferation of hepatocytes was stronger in the LKO mice than in the control mice that survived the surgery as measured by Ki67 staining (Fig. 2f) and expression levels of proregenerative markers (Fig. 2g, h). Similarly, hepatocyte-restricted Suv39h1 deletion alleviated liver injury and promoted liver regeneration in the CCl_4_ injection model (Supplementary Fig. 7).

#### RNA-seq points to a role for Suv39h1 in regulating cell cycling in hepatocytes

**Fig. 3:**
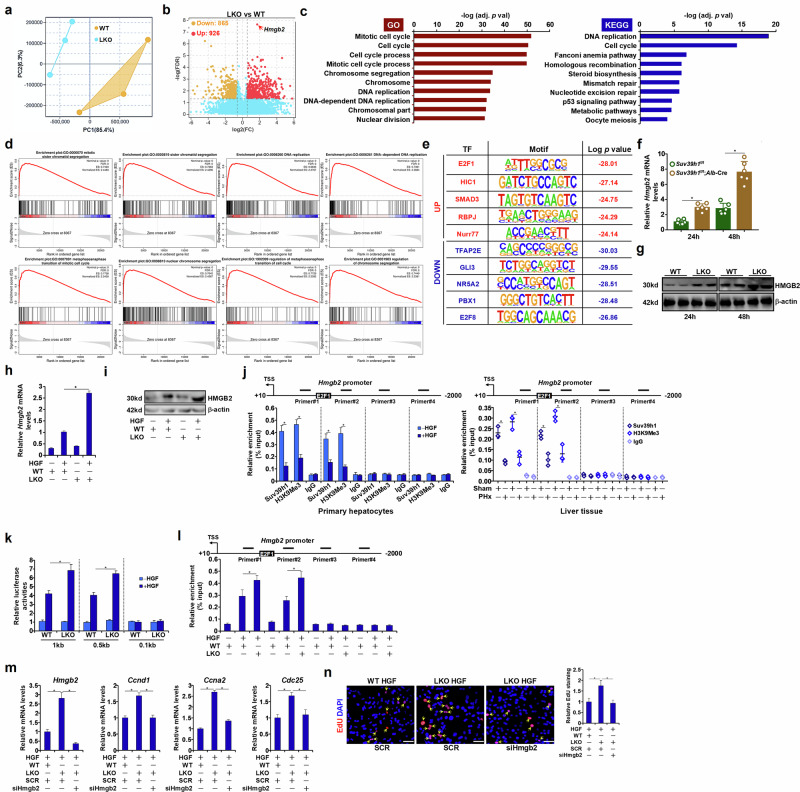
RNA-seq points to a role for Suv39h1 in regulating cell cycling in hepatocytes. **a**–**e** Primary hepatocytes isolated from Suv39h1^LKO^ mice and WT mice were treated with HGF (20 ng/ml) for 24 h. RNA-seq was performed as described in the Methods. The image shows the principle component analysis plot (**a**), volcano plot (**b**), GO and KEGG analysis (**c**), GSEA (**d**) and HOMER analysis (**e**). **f**, **g** Suv39h1^LKO^ mice and WT mice were subjected to 2/3 PHx and killed at indicated time points. HMGB2 expression was examined by qPCR (**f**) and Western blotting (**g**). *N* = 4–6 mice for each group. Data are expressed as mean ± s.d. ^*^P < 0.05, two-tailed Student’s *t*-test. **h**, **i** Primary hepatocytes isolated from Suv39h1^LKO^ mice and WT mice were treated with or without HGF (20 ng/ml) for 24 h. HMGB2 expression was examined by qPCR (**h**) and Western blotting (**i**). *N* = 3 biological replicates. Data are expressed as mean ± s.d. ^*^P < 0.05, one-way ANOVA with post hoc Scheffe’s analyses. **j** ChIP assay was performed with anti-Suv39h1, anti-H3K9Me3 or IgG using primary hepatocytes or liver lysates. *N* = 3 biological replicates (hepatocytes) or three mice (liver tissues) for each group. Data are expressed as mean ± s.d. ^*^P < 0.05, one-way ANOVA with post hoc Scheffe’s analyses. **k** HMGB2 promoter–luciferase constructs were transfected into hepatocytes followed by treatment with HGF (20 ng/ml) for 24 h. Luciferase activity was normalized by protein concentration and green fluorescent protein fluorescence. *N* = 3 biological replicates. Data are expressed as mean ± s.d. ^*^P < 0.05, one-way ANOVA with post hoc Scheffe’s analyses. **l** Primary hepatocytes isolated from Suv39h1^LKO^ mice and WT mice were treated with HGF (20 ng/ml) for 24 h. ChIP assay was performed with anti-E2F1. *N* = 3 biological replicates. Data are expressed as mean ± s.d. ^*^P < 0.05, one-way ANOVA with post hoc Scheffe’s analyses. **m**, **n** Primary hepatocytes isolated from Suv39h1^LKO^ mice and WT mice were transfected with indicated siRNAs followed by treatment with (20 ng/ml) for 24 h. Gene expression levels were examined by qPCR and cell proliferation was evaluated by EdU incorporation. *N* = 3 biological replicates. Data are expressed as mean ± s.d. ^*^P < 0.05, one-way ANOVA with post hoc Scheffe’s analyses.

To gain genome-wide insights for the regulation of liver regeneration by Suv39h1, primary hepatocytes isolated from the LKO mice and the control mice were exposed to HGF treatment followed by RNA-seq. Suv39h1 deficiency profoundly altered the cellular transcriptome, resulting in thousands of differentially expressed genes (Fig. 3a, b). Gene Ontology (GO) pathway analysis and Kyoto Encyclopedia of Genes and Genomes (KEGG) pathway analysis indicated that Suv39h1 deletion predominantly influenced expression of genes involved in cell cycling (Fig. 3c). Gene set enrichment analysis (GSEA) confirmed that Suv39h1 deficiency was positively correlated with proliferative response in hepatocytes (Fig. 3d). Hypergeometric Optimization of Motif Enrichment (HOMER) analysis indicated that Suv39h1 deficiency augmented the activity of several proregenerative transcription factors including E2F1 (Fig. 3e). As Suv39h1 primarily functions as a transcriptional repressor, we focused on the 926 genes up-regulated by Suv39h1 deletion; among these genes, high mobility group family B member 2 (HMGB2) was identified to be the most significantly altered (Fig. 3b).

HMGB2 expression peaked at 24 h but declined to baseline at 72 h after PHx; this kinetic lagged slight behind that of Suv39h1 expression in the same setting (Supplementary Fig. 8). qPCR (Fig. 3f) and Western blotting (Fig. 3g) verified that Suv39h1 deletion further amplified HMGB2 up-regulation. Likewise, Suv39h1 deficiency further enhanced induction of HMGB2 expression by HGF treatment in primary hepatocytes (Fig. 3h, i). In addition, ChIP assay showed that following HGF exposure or PHx surgery, Suv39h1 occupancy was reduced while trimethylated H3K9 was erased from the proximal, but the not distal, HMGB2 promoter (Fig. 3j). Of note, removal of the proximal E2F1 binding site within the HMGB2 promoter abrogated the effect of both HGF and Suv39h1 (Fig. 3k). Indeed, Suv39h1 deletion promoted the recruitment of E2F1 to the HMGB2 promoter (Fig. 3l). Importantly, HMGB2 silencing negated the proliferative advantage of Suv39h1-null hepatocytes when exposed to HGF treatment (Fig. 3m, n). Collectively, these data suggest that HMGB2 may be a novel target for Suv39h1 in the context of liver regeneration.

#### HMGB2 plays an essential role in liver regeneration

**Fig. 4:**
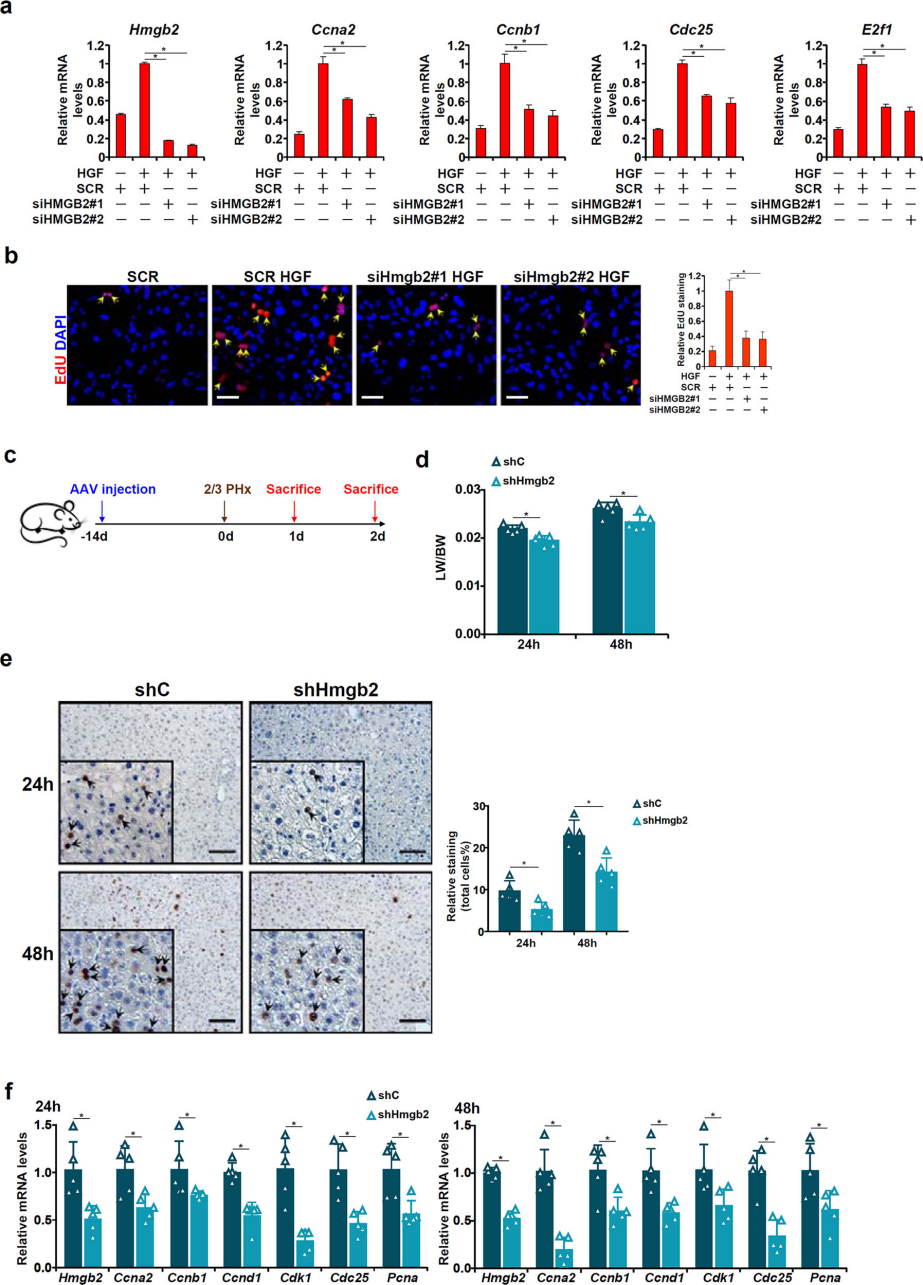
HMGB2 plays an essential role in liver regeneration. **a**, **b** Primary hepatocytes were transfected with siRNA targeting HMGB2 or scrambled siRNA (SCR) followed by treatment with HGF (20 ng/ml) for 24 h. Gene expression levels were examined by qPCR (**a**). Cell proliferation was evaluated by EdU incorporation (**b**). *N* = 3 biological replicates. Data are expressed as mean ± s.d. ^*^P < 0.05, one-way ANOVA with post hoc Scheffe’s analyses. **c**–**f** C57/BL6 mice were injected via the tail vein AAV8 carrying shRNA targeting HMGB2 or a control shRNA followed by PHx (2/3 PHx) and killed at indicated time points. The images show the scheme of the protocol (**c**) and liver weight versus body weight (**d**). Paraffin sections were stained for Ki67. Quantifications were performed with Image Pro (**e**). Gene expression levels were examined by qPCR (**f**). *N* = 5–6 mice for each group. Data are expressed as mean ± s.d. *P < 0.05, two-tailed Student’s *t*-test.

The next series of experiments were performed to determine the functional relevance of HMGB2 in liver regeneration. Silencing of HMGB2 expression in primary hepatocytes by two different pairs of small interfering RNAs (siRNAs) attenuated HGF-induced expression of pro-proliferative genes (Fig. 4a) and cell proliferation (Fig. 4b). Then shRNA targeting HMGB2 was packaged into AAV8 and injected into C57 mice followed by 2/3 PHx to determine how HMGB2 silencing would influence liver regeneration in vivo (Fig. 4c). Western blotting showed that HMGB2 levels were significantly downregulated in the livers of mice injected with AAV-shHmgb2 compared with those injected with AAV-shC (Supplementary Fig. 9). Indeed, HMGB2 depletion markedly decelerated liver regeneration as evaluated by liver weight/body weight ratios (Fig. 4d), Ki67 staining (Fig. 4e) and expression of proregenerative markers (Fig. 4f). Similarly, in the CCl_4_ injection model, HMGB2 silencing exacerbated liver injury and dampened liver regeneration (Supplementary Fig. 10).

#### Integrated transcriptomic analysis provides mechanistic insight and uncovers novel HMGB2 targets

**Fig. 5:**
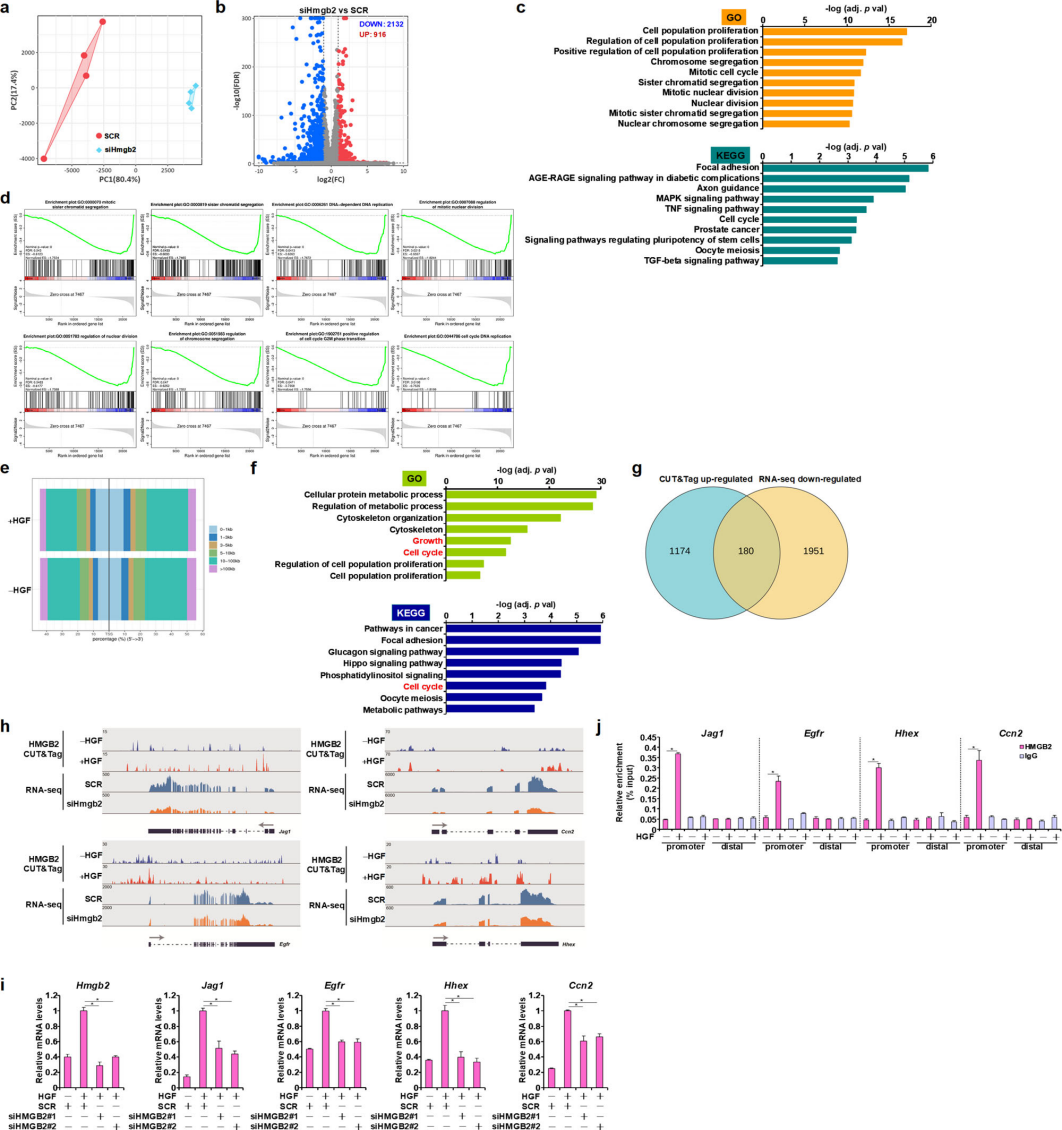
HMGB2 programs proregenerative transcription in hepatocytes. **a**–**d** Primary hepatocytes were transfected with siRNA targeting HMGB2 or scrambled siRNA (SCR) followed by treatment with HGF (20 ng/ml) for 24 h. RNA-seq was performed as described in the Methods. The images show the principle component analysis plot (**a**), volcano plot (**b**), GO and KEGG analysis (**c**) and GSEA (**d**). **e**, **f** Primary hepatocytes were treated with or without HGF (20 ng/ml) for 24 h. CUT&Tag-seq was performed as described in the Methods. The images show the distribution of HMGB2 peaks (**e**) and GO and KEGG analysis (**f**). **g** Venn diagram. **h** CUT&Tag tracks of HMGB2 signals and RNA-seq tracks of the read coverage surrounding the indicated loci. **i** Primary hepatocytes were transfected with siRNA targeting HMGB2 or scrambled siRNA (SCR) followed by treatment with HGF (20 ng/ml) for 24 h. Gene expression was examined by qPCR. **j** Primary hepatocytes were treated with or without HGF (20 ng/ml) for 24 h. ChIP assay was performed with anti-HMGB2 or IgG. *N* = 3 biological replicates. Data are expressed as mean ± s.d. *P < 0.05, one-way ANOVA with post hoc Scheffe’s analyses.

To unveil genome-wide mechanism whereby HMGB2 might regulate liver regeneration, primary hepatocytes were depleted of HMGB2 by siRNA followed by HGF treatment and subsequently RNA-seq. HMGB2 silencing dramatically altered the transcriptome of hepatocytes (Fig. 5a). Many more genes were being up-regulated (2132) than being downregulated (916) as result of HMGB2 silencing (Fig. 5b). GO/KEGG analysis and GSEA indicated that HMGB2, similar to Suv39h1, predominantly regulated genes involved in cell cycling (Fig. 5c) and that the loss of HMGB2 seemed to be associated with defective cell proliferation (Fig. 5d). Next, primary hepatocytes were treated with or without HGF followed by Cleavage Under Targets and Tagmentation followed by sequencing (CUT&Tag-seq) to determine genome-wide binding patterns of HMGB2 in hepatocytes. As shown in Fig. 5e, HGMB2 occupancy on the promoter regions was significantly enhanced by HGF treatment. GO/KEGG analysis of those genes whose promoters were differentially occupied by HMGB2 following HGF treatment indicated that HMGB2 might preferentially bind to cell cycle-related gene promoters (Fig. 5f). A total of 180 genes, whose promoters were inducibly occupied by HMGB2 and whose levels were downregulated by HMGB2 knockdown, were identified in hepatocytes (Fig. 5g). Among these genes were ligands (for example, *Jag1*), receptors (for example, *Egfr*), transcription factors (for example, *Hhex*) and effector molecules (for example, *Ccn2*), indicating that HMGB2 probably contributes to liver regeneration by controlling the threshold of pro-proliferative genes (Fig. 5h). ChIP assay (Fig. 5i) and qPCR assay (Fig. 5j) validated the transcriptomic data.

#### Suv39h1 inhibition promotes liver regeneration mice

**Fig. 6:**
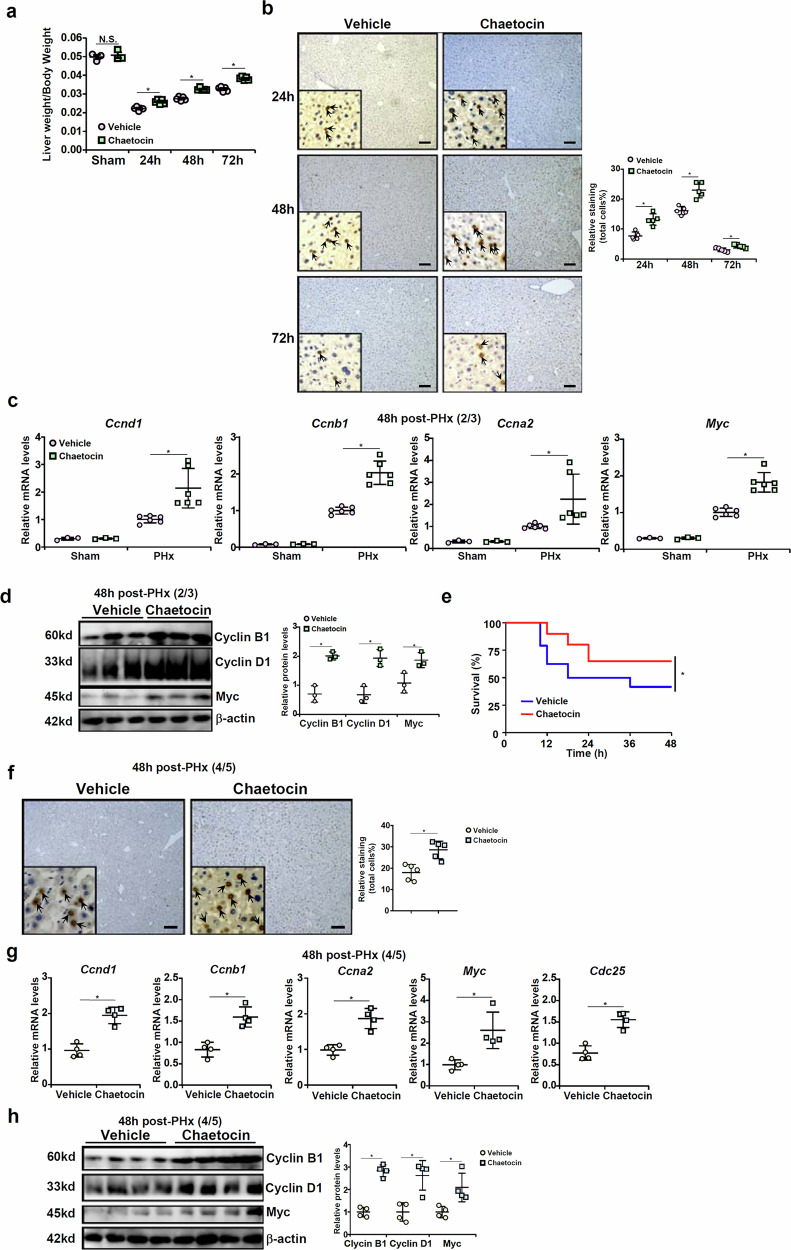
Suv39h1 inhibition promotes liver regeneration mice. **a**–**d** C57/BL6 mice were injected with chaetocin (0.25 mg/kg) daily for 3 days before PHx (2/3 PHx) and killed at indicated time points. The image shows liver weight versus body weight (**a**). Paraffin sections were stained for Ki67. Quantifications were performed with Image Pro (**b**) and gene expression levels were examined by qPCR (**c**) and Western blotting (**d**). *N* = 3 mice for the vehicle groups and *N* = 5 mice for the chaetocin groups. Data are expressed as mean ± s.d. ^*^P < 0.05, two-tailed Student’s *t*-test. **e**–**h** C57/BL6 mice were injected daily with chaetocin (0.25 mg/kg) for 3 days before 4/5 PHx and monitored for 48 h after surgery. The image shows the Kaplan–Meier plot (**e**). *N* = 20 mice for each group. Paraffin sections were stained for Ki67. Quantifications were performed with Image Pro (**f**) and gene expression levels were examined by qPCR (**g**) and Western blotting (**h**). *N* = 5 mice for each group. Data are expressed as mean ± s.d. ^*^P < 0.05, two-tailed Student’s *t*-test.

Next, we sought to determine whether Suv39h1 inhibition by a small-molecule compound (chaetocin)^17^ could be leveraged as an approach to stimulate liver regeneration. To this end, chaetocin was administered via peritoneal injection for three consecutive days before the PHx surgery. Histone H3K9 trimethylation, used as a proxy for Suv39h1 activity, was significantly lower in the primary hepatocytes isolated from the chaetocin-injected mice than the vehicle-injected mice (Supplementary Fig. 11). Suv39h1 inhibition significantly enhanced liver regeneration, as gauged by liver weight/body weight (Fig. 6a), Ki67 staining of proliferating hepatocytes (Fig. 6b) and expression levels of pro-proliferative/regenerative markers (Fig. 6c, d). We also assessed the effect of Suv39h1 inhibition on 4/5 PHx in mice. Consistently, there were fewer post-surgery deaths associated with the mice receiving chaetocin injection compared with the control mice following 4/5 hepatectomy (Fig. 6e). Again, the surviving advantage afforded by chaetocin injection in mice was mirrored by augmented proliferation of hepatocytes (Fig. 6f–h). Of note, chaetocin injection was observed to protect the mice from CCl_4_-induced liver injury and boosted post-injury liver regeneration in mice (Supplementary Fig. 12).

#### Relevance of the Suv39h1–HMGB2 axis in liver regeneration in humans

**Fig. 7:**
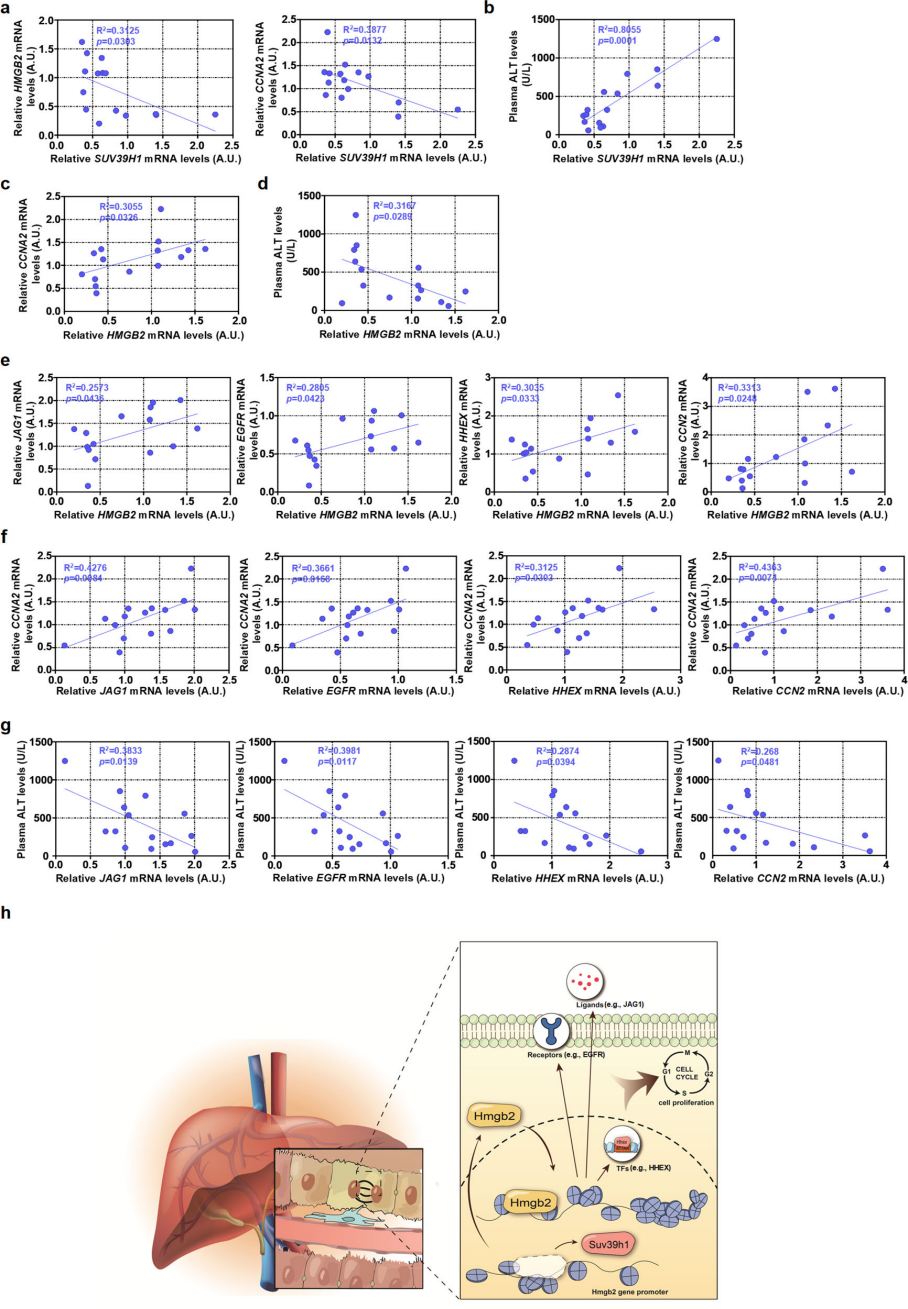
Relevance of SUV39H1 and HMGB2 in liver regeneration in humans. **a** Correlation between *SUV39H1* expression, *HMGB2* expression and *CCNA2* expression in the livers. **b** Correlation between *SUV39H1* expression in the livers and plasma ALT levels. **c** Correlation between *HMGB2* expression and *CCNA2* expression in the livers. **d** Correlation between *HMGB2* expression in the livers and plasma ALT levels. **e** Correlation between *HMGB2* expression and *JAG1*, *EGFR*, *HHEX* and *CCN2* expression in the livers. **f** Correlation of *JAG1*, *EGFR*, *HHEX* and *CCN2* expression with *CCNA2* expression in the livers. **g** Correlation of *JAG1*, *EGFR*, *HHEX* and *CCN2* expression in the livers and plasma ALT levels. *N* = 15 cases. **h** A schematic model summarizing the major finding of this study.

Finally, to find support for a relevance of the newly identified Suv39h1–HMGB2 axis in liver regeneration in humans, liver specimens were collected from a small cohort of patients with acute liver failure. As shown in Fig. 7a, an inverse correlation was identified between *SUV39H1* expression and *HMGB2* expression in the human livers. In addition, *SUV39H1* expression was found to be inversely correlated with proliferation of hepatocytes (*CCNA2* as a proxy; Fig. 7a). Consistently, *SUV39H1* expression was positively correlated with liver injury (plasma ALT level as a proxy; Fig. 7b). On the contrary, *HMGB2* expression was positively correlated with proliferation of hepatocytes (Fig. 7c) but inversely correlated with liver injury (Fig. 7d). Importantly, HMGB2 targets identified by RNA-seq and CUT&Tag-seq, including *JAG1*, *EGFR*, *HHEX* and *CCN2*, were positively correlated with *HMGB2* (Fig. 7e), positively correlated with proliferation of hepatocytes (Fig. 7f) but inversely correlated with liver injury (Fig. 7g). Combined, these data suggest that the Suv39h1–HMGB2 axis may regulate liver regeneration in humans.

## Discussion

Robust liver regeneration offsets liver injury and boosts survival whereas crippled liver regeneration dampens recovery of liver function, rendering the host more vulnerable to liver injury. Here, we detail an epigenetic pathway wherein the histone H3K9 methyltransferase Suv39h1 regulates liver regeneration by programming proliferation-related transcriptional events in hepatocytes (Fig. 7h). We show that transient repression of Suv39h1 coincided with the regenerative response in the liver, which was mediated by DNMT1-mediated hypermethylation of the *Suv39h1* locus in hepatocytes. This was consistent with a previously reported pro-regenerative role of DNMT1. Kaji et al. first reported that DNMT1 deletion in postnatal hepatocytes compromised proliferation by inducing apoptosis and senescence^30^. In a separate study by Wang et al., DNMT1 was found to be significantly up-regulated during liver regeneration right around the time when hepatocytes re-enter the cell cycle^31^. The mechanism whereby Suv39h1 regulates liver regeneration as presented here, we suspect, is far from exhaustive. How Suv39h1 influences global binding patterns of key transcription factors involved in liver regeneration across different cell types remains unsolved and deserves further investigation. First, Suv39h1 might, in addition to relying on HMGB2, directly regulate cell cycle-related genes. For instance, Hung and colleagues have shown that Suv39h1, recruited by sequence-specific transcription factor Sp1, can directly bind to the *Ccnb2* promoter to repress its transcription^32^. In another study, Suv39h1 was found to interact with parafibromin, a component of the polymerase-associated factor 1 (PAF1) complex, to bind to the *Ccnd1* promoter and repress *Ccnd1* transcription^33^. Second, we focused on the regulation of hepatocyte proliferation as a proxy for evaluating the role of Suv39h1 in liver regeneration. There are alternative aspects that deserve attention. Excessive reactive oxygen species (ROS) correlates with decelerated liver regeneration as evidenced by the observation that mice with a deficiency in Nrf2, a potent antioxidant transcription factor, exhibit retarded recovery from PHx^34^. Interference with mitochondrial function, where a majority of ROS is produced, also results in impairment of liver regeneration^35^. Suv39h1 has previously been shown to regulate intracellular ROS levels^18,36^. Thus, our observation that Suv39h1 deficiency improves liver regeneration can be equally attributed to normalization of hepatic redox status. Similarly, accumulating evidence suggests that proliferation of hepatocytes in the regenerating liver parallels profound skewing of hepatic metabolism characterized by hypoglycemia and transient steatosis^37^. Several independent reports have previously shown that Suv39h1 activity can be fine-tuned by extracellular metabolic cues or energy status^38,39^. In addition, Suv39h1 is a direct transcriptional repressor of SIRT1, the master regulator of cellular metabolism^17^. It would be of great interest to determine whether regulation of liver regeneration by Suv39h1 is contingent upon metabolic reprogramming.

Although we made an attempt to correlate locus-specific histone modification and transcription outcome to assess Suv39h1 activity, the possibility that Suv39h1 may regulate liver regeneration via lysine methylation of non-histone factors cannot be ignored. Thus far, few non-histone Suv39h1 substrates have been identified and functionally analyzed in the context of liver regeneration. A report by Kim et al. suggests that lysine methylation of the transcription factor RUNX2 by Suv39h1 inhibits its activity probably owing to altered affinity for target promoters^40^. A direct role for RUNX2 in liver regeneration is lacking, and several pro-proliferative genes including *c-Myc* can be directly activated by RUNX2^41^. Thus, Suv39h1 deficiency may emancipate sequence-specific transcription factors (for example, RUNX2) that remain dormant under normal conditions to promote proliferation of hepatocytes and participate in liver regeneration.

Through RNAs-seq screening, we identify HMGB2 as a direct target of Suv39h1. Further, integrated transcriptomic analysis illustrates that loss of Suv39h1 allows derepression of HMGB2, which in turn orchestrates a proregenerative transcriptional program to promote liver regeneration. Of note and contrary to our finding, Huang et al. have found that the Hmgb2^−/−^ mice display reduced liver injury compared with WT littermates receiving CCl_4_ injection^42^. This discrepancy can be explained by the fact that HMGB2 is a strong proinflammatory factor in myeloid and lymphoid cells^43,44^. Our observation echoes a recently published study by Choijookhuu et al. in which the authors show that systemic deletion of HMGB2 (Hmgb2^−/−^) dampens liver regeneration in mice^45^. Instead of relying on the (outdated) micro-array technique by the Choijookhuu et al. study, we used a combination of RNAs-seq and CUT&Tag-seq to mine for potential HMGB2 targets and to decipher the mechanism whereby HMGB2 promotes liver regeneration. Among the novel HMGB2 targets uncovered by this strategy, many are well-established regulators of liver regeneration that include Jag1^46^, Egfr^47^, Hhex^48^ and Ccn2^49^. The pro-regenerative potential of HMGB2 has been highlighted by a string of recent discoveries that portray HMGB2 as a marker for brain intermediate progenitor cells^50^, muscle satellite cells^51^ and endometrial clonogenic cells^52^. Thus, boosting HMGB2 activity may be considered as a reasonable approach to resuscitate a failing liver.

The most intriguing finding of this study is that pharmaceutical inhibition of Suv39h1 promotes liver regeneration while limiting liver injury. Dampened liver regeneration, or absence of Ki67^+^ hepatocytes as highlighted in two recent studies, prevents effective recovery from and contributes to poor prognosis in end-stage liver diseases including cirrhosis^53,54^. In this context, it is worth noting that our recent publication indicates that chaetocin administration attenuates liver fibrosis in mice^20^. However, defective liver regeneration is thought to promote regrowth of hepatocellular carcinoma following surgical resection^55^. It is worth noting that it has been previously demonstrated that chaetocin administration antagonizes the development of hepatocellular carcinoma in mice^56^. These data highlight the potential versatility of Suv39h1 in regulating physiological and pathological proliferation of hepatocytes, as key differences exist between these processes. Therefore, targeting Suv39h1 with small-molecule inhibitors in clinical settings would probably achieve multiple benefits. However, the mechanisms that underscore the differentiation between the anti-regenerative and the pro-oncogenic roles of Suv39h1 in the liver remain unclear at present. It should be noted that Suv39h1 expression is quickly but transiently altered (within 12 h following PHx) during liver regeneration. However, previous studies suggest that alteration of Suv39h1 expression can only be detected long after the initial oncogenic stimuli. For instance, Takeuchi et al. have shown that Suv39h1 expression is up-regulated in the humanized mouse livers more than 7 months after infection with hepatitis B virus in a model of hepatitis B virus-associated hepatocellular carcinoma^57^. Pogribny et al. have reported that in a rat model of methyl-deficient diet induced hepatocellular carcinoma Suv39h1 expression in the liver does not significantly change until after 36 weeks of feeding^58^. It is tempting to speculate that short-term transient alterations in Suv39h1 may be associated with compensatory or beneficial response (for example, liver regeneration), whereas long-term persistent alterations in Suv39h1 may lead to maladaptive or detrimental consequences (for example, carcinogenesis). Alternatively, post-translational modifications of Suv39h1 by SIRT1, which deacetylates lysine 266 of Suv39h1^59^, CDK2, which phosphorylates serine 391 of Suv39h1^59^ and SET7/9, which methylates lysines 105 and 123 of Suv39h1^60^, differentially regulate Suv39h1 activity. Therefore it is plausible that distinctive post-translational modification status, in response to a specific cue, may ‘barcode’ Suv39h1 to execute a specific functionality (for example, regeneration or oncogenesis). Clearly, additional investigations are warranted to disentangle these possibilities.

Despite the advances of our study that highlight Suv39h1 as an actionable target for the intervention of liver failure, there are significant limitations that may dampen its translational potential. First, all the transcriptomic sequencing experiments were performed with primary hepatocytes, which tend to dedifferentiate when cultured ex vivo. Future investigations that attempt to tackle this issue by performing experiments with liver tissues either at bulk level or, more ideally, at single-cell level, would supplement and hopefully further validate the data presented here. Second, the Suv39h1–HMGB2 axis appears to be key to liver regeneration in both the PHx model and the CCl_4_ model despite the fact that there are key differences regarding initiating factors in promoting cell proliferation and initial cells involved in these two models. A tempting proposal would be that this newly identified axis might serve as a bridge that links different cellular and molecular pathways to coordinate liver regeneration in different settings. More sophisticated and advanced tools (for example, single-cell spatial transcriptomics/proteomics) will probably provide mechanistic insights for this intriguing issue. Third, despite our proof-of-concept data that the Suv39h1 inhibitor chaetocin could promote liver regeneration in murine models, the toxicological and pharmacological profiles of this chemical remain unappreciated, rendering its applicability in humans untenable, at least for now. In addition, the specificity of chaetocin toward Suv39h1 has been challenged^61,62^. Our data therefore can only be considered as a template for future quest to design more specific Suv39h1-targeting compounds for translational hepatology. Finally, although our finding was validated in human specimens, the sample size was relatively small. To present SUV39H1 as a druggable target for clinical translation, it would be ideal to include larger cohorts of different populations.

In summary, we present evidence here that the histone H3K9 methyltransferase Suv39h1 contributes to liver regeneration probably through unlocking HMGB2 transcription. A comprehensive examination of Suv39h1-dependent function using transcriptomic, metabolomic and proteomic tools will hopefully uncover the full mechanism whereby Suv39h1 contributes to liver regeneration, further our understanding of epigenetic regulation of liver diseases and lead to the development of novel therapeutic solutions.

## Supplementary information

Supplementary Information

## Data Availability

The sequencing data generated by this study have been uploaded in the PubMed GEO repository. The accession numbers are, for the RNA-seq shown in Fig. 3, GSE313425; for the RNA-seq shown in Fig. 5, GSE313198; and for the CUT&Tag-seq shown in Fig. 5, GSE313427.
