# Supplementary material for: Genetic and pharmaceutical manipulation of H3K9 methyltransferase Suv39h1 promotes liver regeneration by unleashing HMGB2 transcription
Source: Exp Mol Med. 2026 Apr 10;58(4):1158–71. doi: 10.1038/s12276-026-01677-4 (PMC13144335; doi:10.1038/s12276-026-01677-4)
Supplement: Supplementary file 1 — Supplementary Information [file 12276_2026_1677_MOESM1_ESM.pdf]

**Lu Y et al: Genetic and pharmaceutical manipulation of H3K9 methyltransferase Suv39h1 promotes liver regeneration by unleashing HMGB2 transcription**

**Online supplementary material**

**Supplementary Methods: 1**

**Supplementary figures: 12**

**Methods**

*RNA Isolation and Real-time PCR*

RNA was extracted with the RNeasy RNA isolation kit (Qiagen, 74104). Reverse transcriptase reactions were performed using a SuperScript First-strand Synthesis System (Thermo Fisher, 18080093). Real-time PCR reactions were performed on an ABI Prism 7500 system with the following primers: mouse *Suv39h1*, 5’-CTGTGCCGACTAGCCAAGC-3’ and 5’-ATACCCACGCCACTTAACCAG-3’; human *SUV39H1*, 5’-CCTGCCCTCGGTATCTCTAAG-3’ and 5’-ATATCCACGCCATTTCACCAG-3’; mouse *Ccna2*, 5’-AAGAGAATGTCAACCCCGAAAAA-3’ and 5’-ACCCGTCGAGTCTTGAGCTT-3’; human *CCNA2*, 5’-CGCTGGCGGTACTGAAGTC-3’ and 5’-GAGGAACGGTGACATGCTCAT-3’; mouse *Ccnd1*, 5’-GCGTACCCTGACACCAATCTC-3’ and 5’-ACTTGAAGTAAGATACGGAGGGC-3’; mouse *Pcna*, 5’-TTTGAGGCACGCCTGATCC-3’ and 5’-GGAGACGTGAGACGAGTCCAT-3’; mouse *Myc*, 5’-CTTCTCTCCGTCCTCGGATTCT-3’ and 5’-GAAGGTGATCCAGACTCTGACCTT-3’; mouse *Cdc25*, 5’-GCAGCAGCGTTAATTCATCTACT-3’ and 5’-GGCCGAAGAGAGTTTGTCCAC-3’; mouse *Ccnb1*, 5’-CAATTATCGGAAGTGTCGGATCA-3’ and 5’-CTGGTGAACGACTGAACTCCC-3’; human *HMGB2*, 5’-CGGGGCAAAATGTCCTCGTA-3’ and 5’-CGGAAGAGTCCGGGTGTTT-3’; mouse *Hmgb2*, 5’-CGGGGCAAAATGTCCTCGTA-3’ and 5’-ATGGTCTTCCATCTCTCGGAG-3’; human *JAG1*, 5’-GTCCATGCAGAACGTGAACG-3’ and 5’-GCGGGACTGATACTCCTTGA-3’; mouse *Jag1*, 5’-ATGCAGAACGTGAATGGAGAG-3’ and 5’-GCGGGACTGATACTCCTTGAG-3’; human *EGFR*, 5’-AGGCACGAGTAACAAGCTCAC-3’ and 5’-ATGAGGACATAACCAGCCACC-3’; mouse *Egfr*, 5’-GCCATCTGGGCCAAAGATACC-3’ and 5’-GTCTTCGCATGAATAGGCCAAT-3’; human *HHEX*, 5’-ACGCCCTTTTACATCGAGGAC-3’ and 5’-CGTGTAGTCGTTCACCGTC-3’;mouse *Hhex*, 5’-CGGACGGTGAACGACTACAC-3’ and 5’-CTTCTCCAGCTCGACGGTC-3’; human *CCN2*, 5’-CAGCATGGACGTTCGTCTG-3’ and 5’-AACCACGGTTTGGTCCTTGG-3’; mouse *Ccn2*, 5’-GGCCTCTTCTGCGATTTCG-3’ and 5’-GCAGCTTGACCCTTCTCGG-3’. Ct values of target genes were normalized to the Ct values of housekeekping control gene (18s, 5’-CGCGGTTCTATTTTGTTGGT-3’ and 5’-TCGTCTTCGAAACTCCGACT-3’ for both human and mouse genes) using the ΔΔCt method and expressed as relative mRNA expression levels compared to the control group which is arbitrarily set as 1.

*Human ALF Specimens*

Liver biopsies were collected from patients with ALF referring to Nanjing Drum Tower Hospital. Written informed consent was obtained from subjects or families of liver donors. All procedures that involved human samples were approved by the Ethics Committee of the Nanjing Drum Tower Hospital and adhered to the principles outlined in the Declaration of Helsinki. Paraffin sections were stained with indicated antibodies. Detailed patient information is summarized in Supplementary Table I.

*EdU Incorporation Assay*

5-ethynyl-2'-deoxyuridine (EdU) incorporation assay was performed in triplicate wells with a commercially available kit (Thermo Fishe, C10337). Briefly, the EdU solution was diluted with the culture media and added to the cells for an incubation period of 2h at 37$^{o}$C. After several washes with 1XPBS, the cells were then fixed with 4% formaldehyde and stained with Alexa Fluor™ 488. The nucleus was counter-stained with DAPI. The images were visualized by fluorescence microscopy and analyzed with Image-Pro Plus (Media Cybernetics). For each well six different fields were randomly chosen and the positively stained cells were counted and divided by the number of total cells. The average of the six fields for each well was calculated. The averages of each group were then normalized to the averages of the control group. The data are expressed as relative EdU staining compared to the control group arbitrarily set as 1.

*Chromatin Immunoprecipitation (ChIP)*

ChIP assays were performed essentially as described before[1-4]. Briefly, chromatins were cross-linked with 1% formaldehyde (Sigma, 11-0735) for 8 min at room temperature and fragmented into ~500bp pieces using a Branson 250 sonicator. 100μg formaldehyde cross-linked nuclear proteins were precipitated with anti-trimethyl H3K9 (Millipore, 07-442), anti-Suv39h1 (Genetex, GTX112263), anti-HMGB2 (Novus Biologicals, NBP3-16774), anti-DNMT1 (Cell Signaling Tech, 5032), anti-5-methylcytosine (Abcam, ab10805), or IgG. Precipitated DNA-protein complexes were washed with RIPA buffer (50mM Tris, pH8.0, 150mM NaCl, 0.1% SDS, 0.5% deoxycholate, 1% NP-40, 1mM EDTA), high salt buffer (50mM Tris, pH8.0, 500mM NaCl, 0.1% SDS, 0.5% deoxycholate, 1% NP-40, 1mM EDTA), LiCl buffer (50mM Tris, pH8.0, 250mM LiCl, 0.1% SDS, 0.5% deoxycholate, 1% NP-40, 1mM EDTA), and TE buffer (10mM Tris, 1mM EDTA pH 8.0), respectively, on a 360 $^{o}$C rotator at 4 $^{o}$C. DNA-protein crosslink was reversed by heating the samples to 65$^{o}$C overnight. Proteins were digested with 0.25 μg/μl Proteinase K (Sigma, P2308) and the released genomic DNA was phenol-chloroform extracted and precipitated by 100% ethanol.Precipitated genomic DNA was amplified by real-time PCR. Serially diluted genomic DNA extracted from normal cells/tissues was used to generate a standard curve to calculate the amount of DNA being precipitated by a particular antibody. A total of 10% of the starting material is also included as the input. Data are then normalized to the input and expressed as fold changes (relative enrichment) compared to the control group.

*RNA Sequencing and Data Analysis*

Total RNA was extracted using the TRIzol reagent according to the manufacturer's protocol. RNA purity and quantification were evaluated using the NanoDrop 2000 spectrophotometer (Thermo Scientific, USA). RNA integrity was assessed using the Agilent 2100 Bioanalyzer (Agilent Technologies, Santa Clara, CA, USA). Then the libraries were constructed using TruSeq Stranded mRNA LT Sample Prep Kit (Illumina, San Diego, CA, USA) according to the manufacturer's instructions and sequenced on an Illumina HiSeq X Ten platform and 150 bp paired-end reads were generated. Raw data (raw reads) of fastq format were firstly processed using Trimmomatic and the low quality reads were removed to obtain the clean reads. The clean reads were mapped to the mouse genome

(Mus_musculus.GRCm38.99) using HISAT2. FPKM of each gene was calculated using Cufflinks, and the read counts of each gene were obtained by HTSeqcount. Differential expression analysis was performed using the DESeq (2012) R package. P value < 0.05 and fold change >1.5 was set as the threshold for significantly differential expression. Hierarchical cluster analysis of differentially expressed genes (DEGs) was performed to demonstrate the expression pattern of genes in different groups and samples. GO enrichment and KEGG pathway enrichment analysis of DEGs were performed respectively using R based on the hypergeometric distribution.

*CUT&Tag Sequencing and Data Analysis*

CUT&Tag assay was performed per vendor recommenations (Vazyme; cat#TD904). For each CUT&Tag experiment, $1 \times 10^5$cells were incubated with 10 µl of ConA Beads. Primary antibody against HMGB2 (Novus Biologicals; cat#NBP3-16774) was then incubated overnight with the chromatin at 4 °C on a shaking platform. Beads-chromatin-antibody mixture was washed once with 200 µl of Dig-wash Buffer, resuspended in 50 µl of Dig-wash Buffer with a mouse secondary antibody, and incubated for 30 min at room temperature on a rotator. The mixture washed twice with 200 µl of Dig-Wash Buffer and resuspended in 100 µl of Dig-300 Buffer containing 2 µl pA/G-Tnp Pro (protein A–Tn5 transposase fusion protein). After incubation with pA/G-Tnp Pro on a rotator at room temperature, the mixture was washed three times with 200 µl Dig-300 Buffer to remove unbound pA/G-Tnp Pro, and resuspended with 50µl Tagmentation buffer. Subsequently, the reaction was stopped with 2 µl 10% SDS and 1 pg DNA Spike-in. DNA was extracted with phenol–chloroform and ethanol. Libraries were amplified according to manufacturer’s instructions. Library quality was evaluated using agarose gel electrophoresis. The libraries were sequencedon an Illumina HiSeq 2500.Quality filtered reads were mapped to the reference genome(GRCm39) using Bowite2. Masc2 was used to call peaks. Deeptoolsand Integrative Genomics Viewer (IGV) were used to accomplish data visualization.

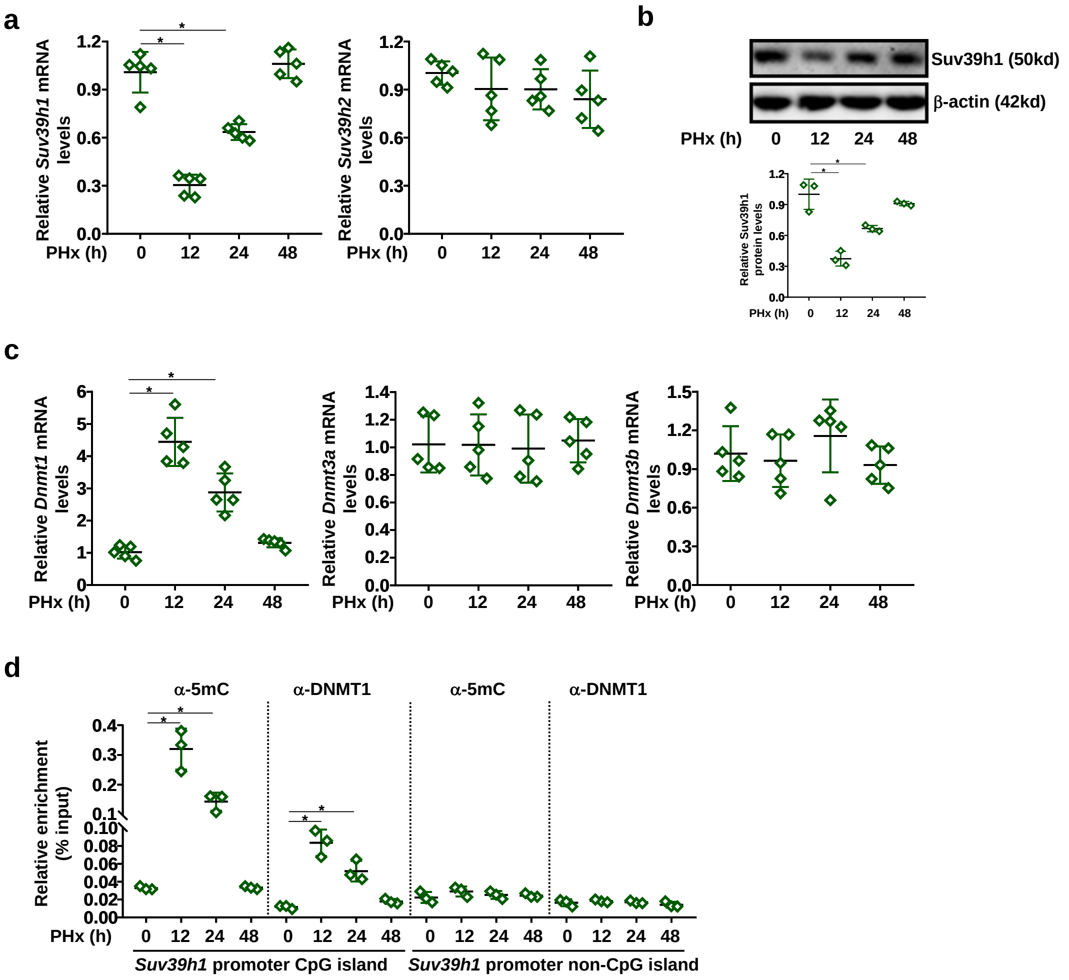


**Supplementary Fig. 1**: (**a, b**) C57/BL6 mice were subjected to 2/3 PHx and sacrificed at indicated time points post-surgery. Suv39h1 expression levels were examined by qPCR and Western blotting. (**c**) C57/BL6 mice were subjected to 2/3 PHx and sacrificed at indicated time points post-surgery. DNMT expression levels were examined by qPCR. (**d**) C57/BL6 mice were subjected to 2/3 PHx and sacrificed at indicated time points post-surgery. ChIP assays were performed with indicated antibodies. N=3-5 mice for each group. Data are expressed as mean±S.D. *, $p<0.05$, one-way ANOVA with post-hoc Scheffe´s.

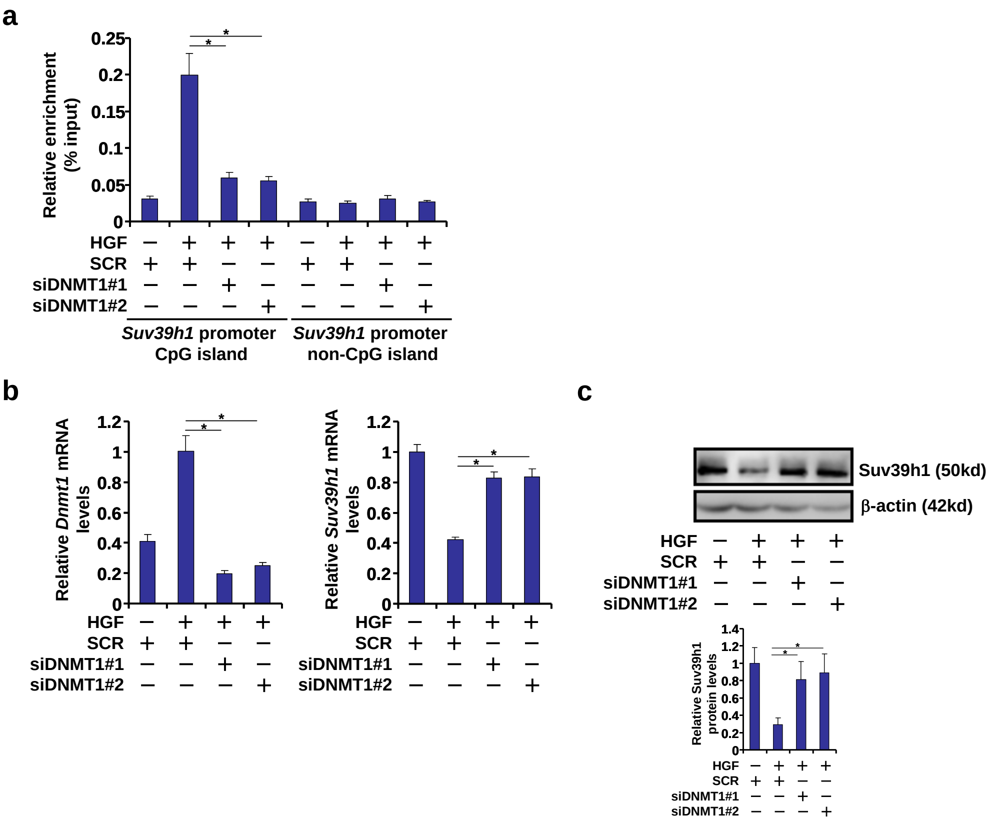


**Supplementary Fig. 2**: (**a-c**) Primary murine hepatocytes were transfected with indicated siRNAs followed by treatment with HGF for 12h. ChIP assay was performed with anti-5-methylcytosine (a). Suv39h1 expression was examined by qPCR (b) and Western blotting (c). N=3 biological replicates. Data are expressed as mean±S.D. *, $p < 0.05$, one-way ANOVA with post-hoc Scheffe´s.

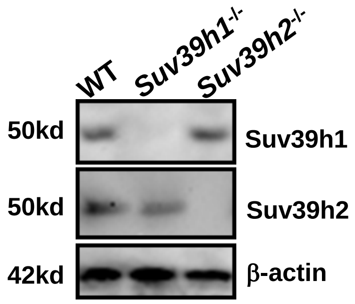


**Supplementary Fig. 3**: Suv39h1 and Suv39h2 expression levels were examined by Western blotting with liver lysates from the *Suv39h1*$^{-/-}$ mice, *Suv39h2*$^{-/-}$ mice, and wild type control mice.

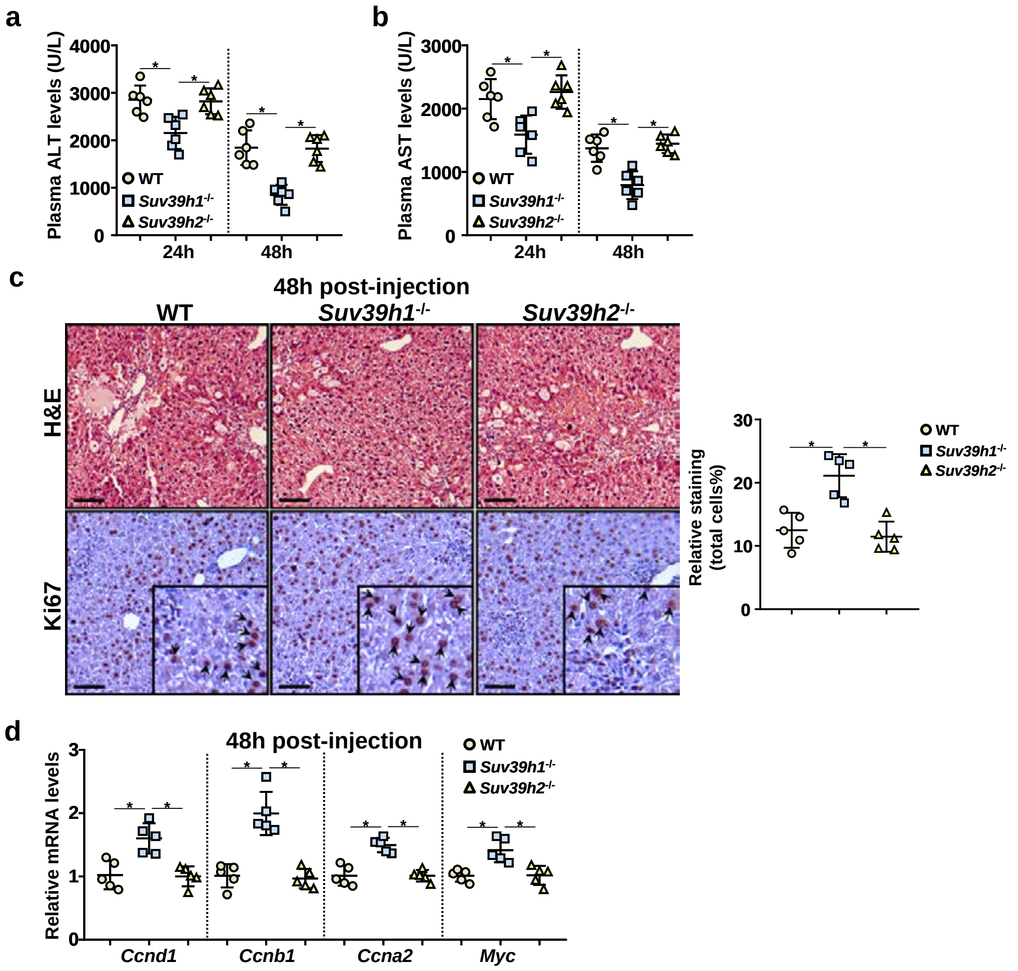


**Supplementary Fig. 4**: *Suv39h1*$^{-/-}$ mice, *Suv39h2*$^{-/-}$ mice and WT mice were injected [illegible]h $CCl_4$and sacrificed at indicated time points. (**a**) Plasma ALT levels. (**b**) Plasma AST levels. (**c**) H&E staining and Ki67 staining. (**d**) Regenerative markers were examined by qPCR at 48h. N=5-6 mice for each group. Data are expressed as mean±S.D. *, $p < 0.05$, one-way ANOVA with post-hoc Scheffe´s.

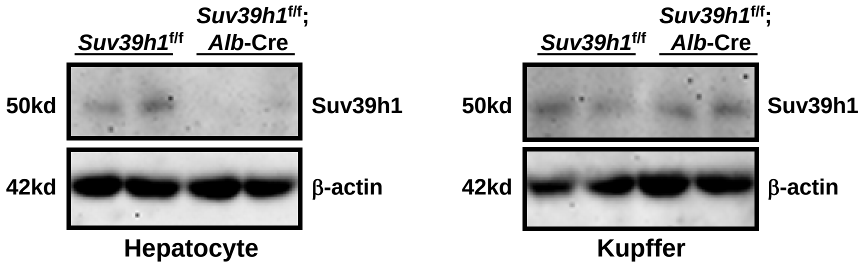


**Supplementary Fig. 5**: Primary hepatocytes and Kupffer cells were isolated from the Suv39h1$^{LKO}$ mice and WT mice. Suv39h1 expression was examined by Western blotting.

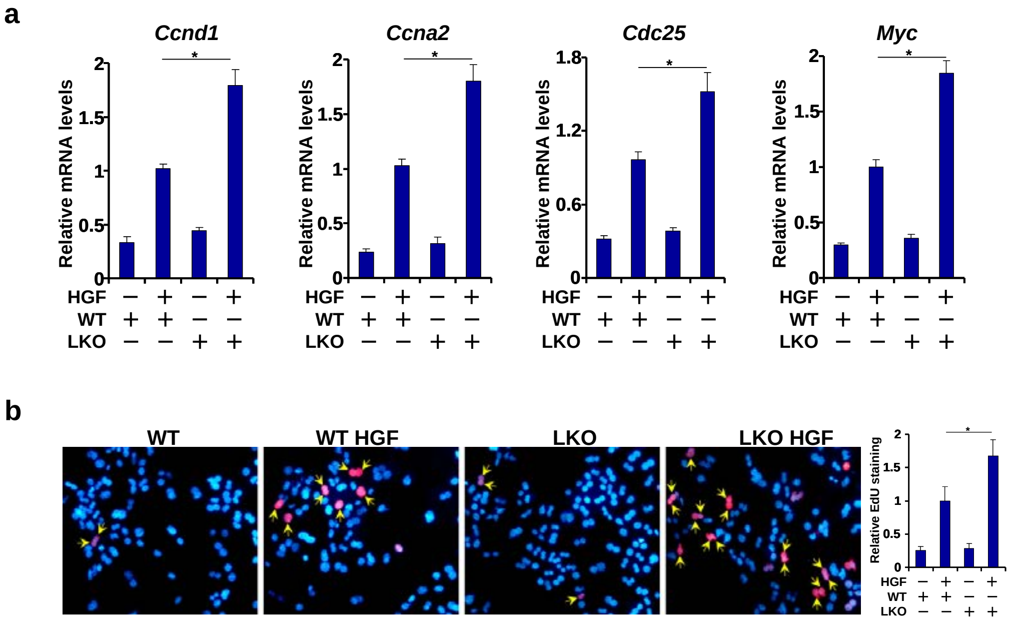


**Supplementary Fig. 6**: Primary hepatocytes were isolated from the Suv39h1$^{LKO}$ mice and WT mice followed by treatment with HGF for 24h. (**a**) Gene expression levels were examined by qPCR. (**b**) Cell proliferation was evaluated by EdU incorporation. N=3 biological replicates. Data are expressed as mean±S.D. *, $p < 0.05$, one-way ANOVA with post-hoc Scheffe´s.

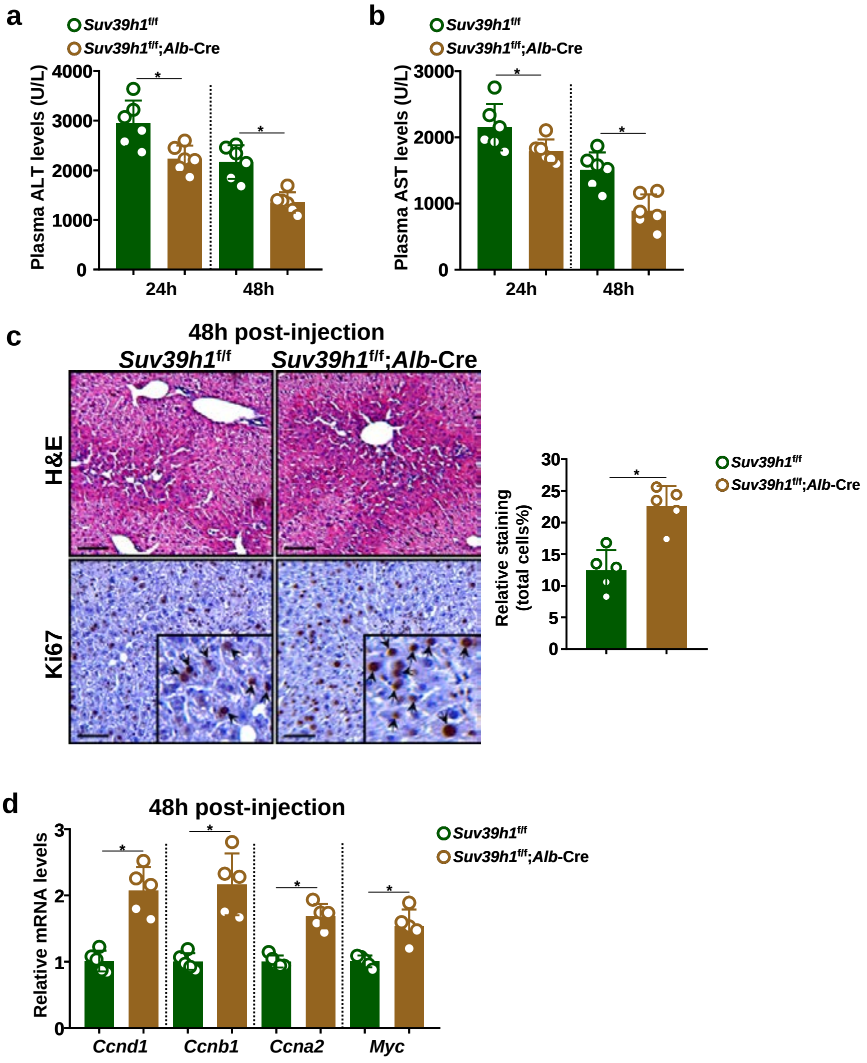


**Supplementary Fig. 7**: Suv39h1$^{LKO}$ mice and WT mice were injected with $CCl_4$and sacrificed at indicated time points. (**a**) Plasma ALT levels. (**b**) Plasma AST levels. (**c**) H&E staining and Ki67 staining. (**d**) Regenerative markers were examined by qPCR. N=5-6 mice for each group. Data are expressed as mean±S.D. *, $p < 0.05$, two-tailed student's test.

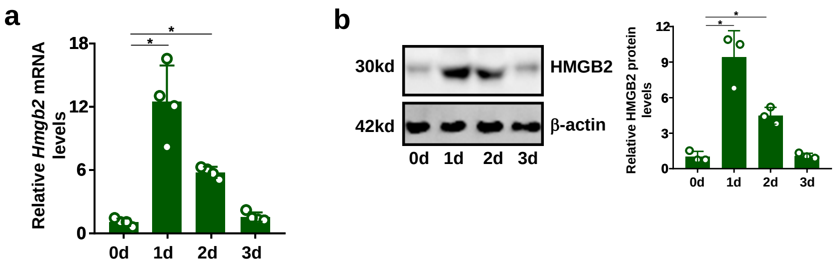


**Supplementary Fig. 8**: (**a, b**) C57/BL6 mice were subjected to 2/3 PHx and sacrificed at indicated time points post-surgery. Hmgb2 expression levels were examined by qPCR and Western blotting. N=3-4 mice for each group. Data are expressed as mean±S.D. *, $p < 0.05$, one-way ANOVA with post-hoc Scheffe´s.

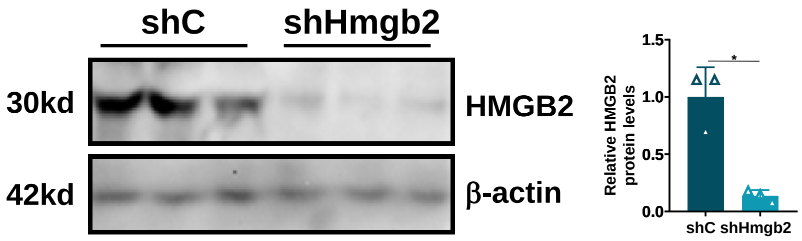


**Supplementary Fig. 9**: C57/BL6 mice were injected via tail vein AAV8 carrying shRNA targeting HMGB2 or a control shRNA. HMGB2 expression levels in the liver was examined by Western blotting. N=3 mice for each group. Data are expressed as mean±S.D. *, $p < 0.05$, two-tailed student's test.

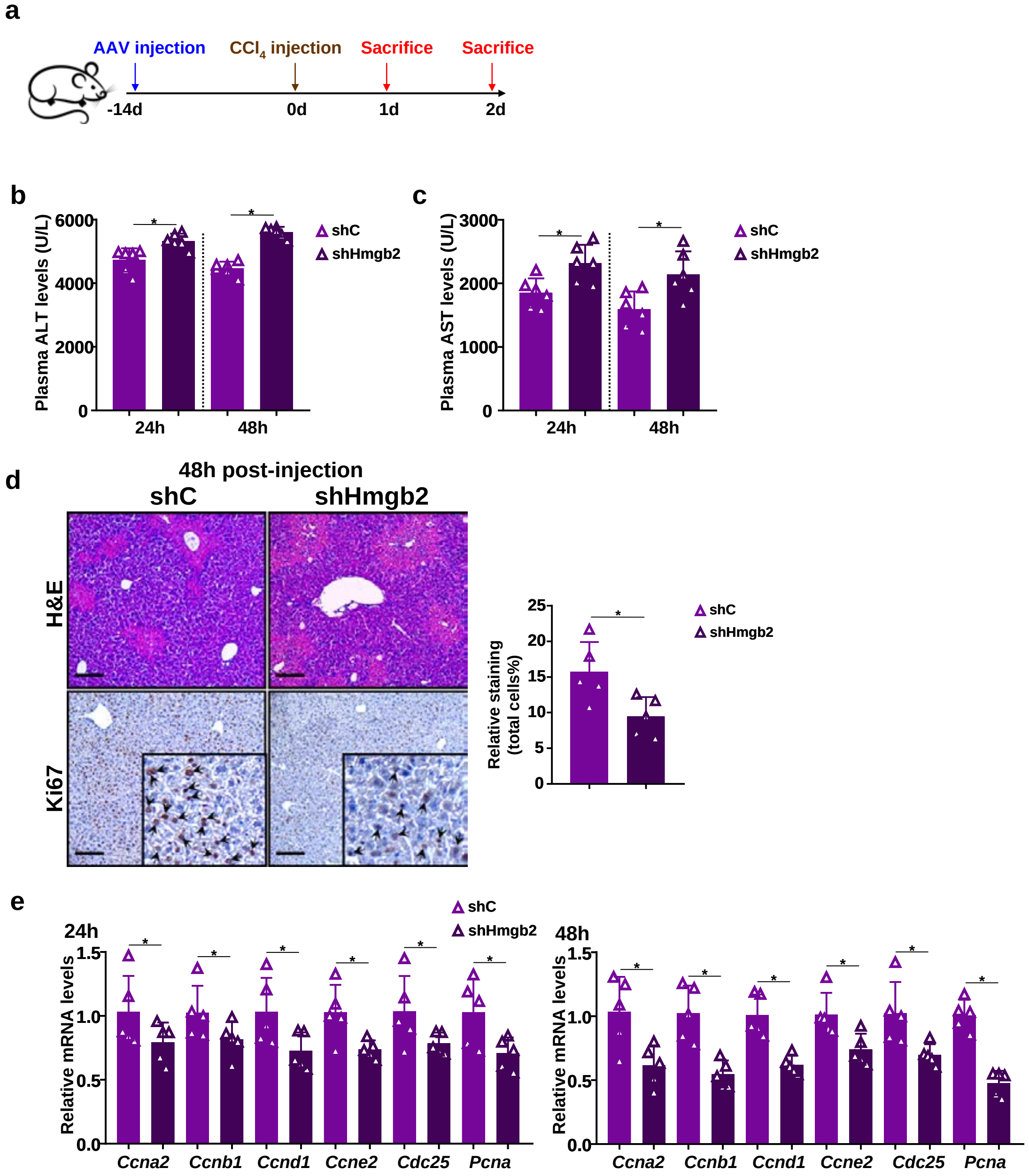


**Supplementary Fig. 10**: C57/BL6 mice were injected via tail vein AAV8 carrying shRNA targeting HMGB2 or a control shRNA followed by $CCl_4$ injection and sacrificed at indicated time points. (**a**) Scheme of protocol. (**b**) Plasma ALT levels. (**c**) Plasma AST levels. (**d**) H&E staining and Ki67 staining. (**e**) Regenerative markers were examined by qPCR. N=5-6 mice for each group. Data are expressed as mean±S.D. *, $p < 0.05$, two-tailed student's test.

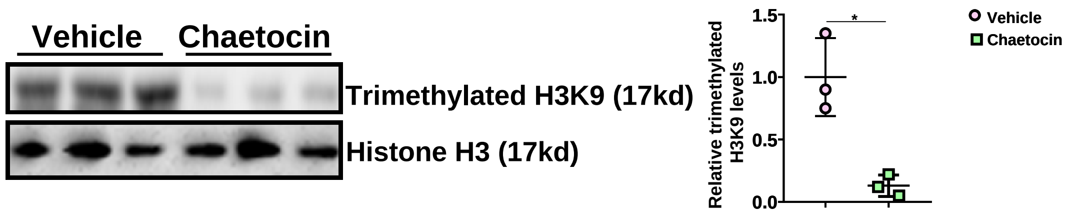


**Supplementary Fig. 11**: C57/BL6 mice were injected with chaetocin (0.25mg/kg) daily for three days prior to 2/3 PHx. Primary hepatocytes were isolated and H3K9Me3 levels were examined by Western blotting.N=3 mice for each group. Data are expressed as mean±S.D. *, $p<0.05$, two-tailed student's test.

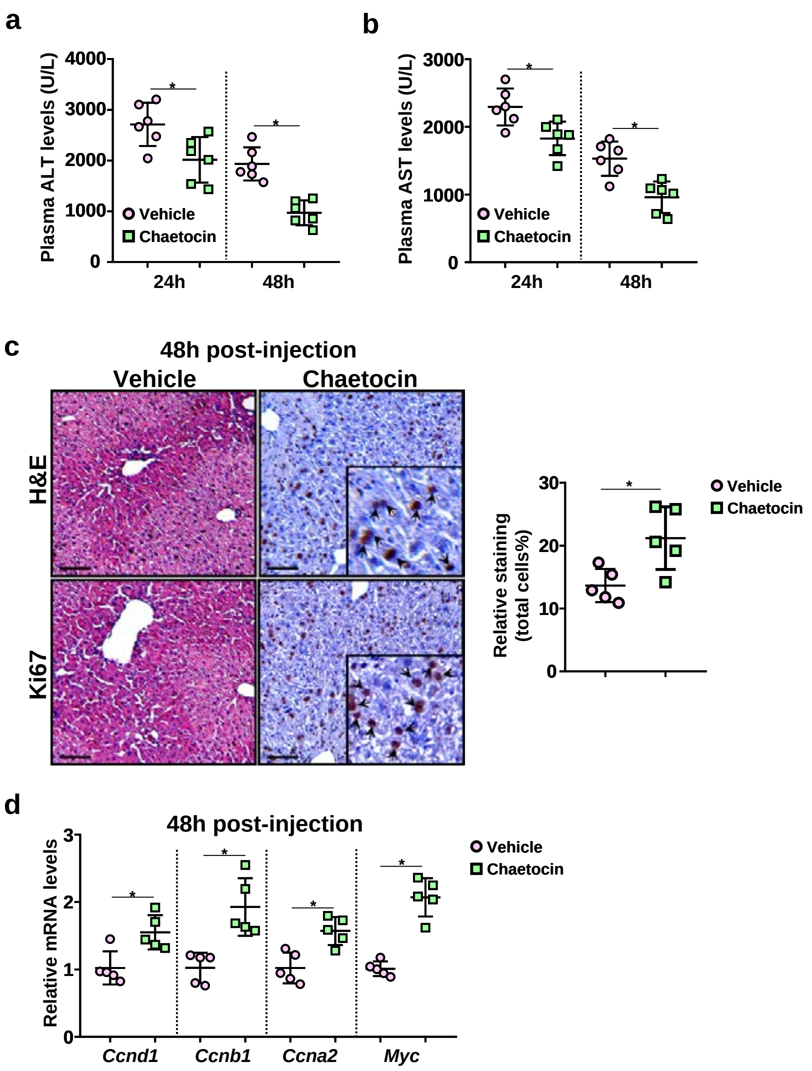


**Supplementary Fig. 12**: C57/BL6 mice were injected with chaetocin (0.25mg/kg) daily for three days prior to $CCl_4$ injection and sacrificed at indicated time points. (**a**) Plasma ALT levels. (**b**) Plasma AST levels. (**c**) H&E staining and Ki67 staining. (**d**) Regenerative markers were examined by qPCR. N=5-6 mice for each group. Data are expressed as mean±S.D. *, $p < 0.05$, two-tailed student's test.

**Supplementary Table I: ALT Patient Information Sheet**

| Patient ID | Gender | Age (yr) | ALT(U/dL) | AST (U/dL) | LDH (U/dL) | Bilirubin(μM) |
|---|---|---|---|---|---|---|
| 1 | Female | 59 | 557.5 | 67.7 | 300 | 154 |
| 2 | Male | 43 | 536.6 | 100.3 | 106 | 217.5 |
| 3 | Male | 51 | 155.1 | 97.6 | 296 | 141 |
| 4 | Male | 41 | 325.4 | 176.8 | 614 | 240 |
| 5 | Male | 51 | 168.2 | 266.1 | 373 | 110.5 |
| 6 | Male | 44 | 109 | 104.8 | 241 | 108.9 |
| 7 | Male | 72 | 791.8 | 713.3 | 390 | 87.6 |
| 8 | Male | 40 | 1247.3 | 1323.6 | 703 | 140.3 |
| 9 | Male | 52 | 56.8 | 66.9 | 464 | 105.2 |
| 10 | Male | 43 | 637.7 | 315.5 | 243 | 143.5 |
| 11 | Male | 47 | 247.9 | 313.4 | 328 | 135.3 |
| 12 | Male | 49 | 851.1 | 869.1 | 490 | 65 |
| 13 | Male | 51 | 264.5 | 411.5 | 614 | 27.4 |
| 14 | Male | 47 | 323.7 | 348.2 | 833 | 106.4 |
| 15 | Male | 36 | 94.9 | 505.6 | 329 | 31.2 |

## References


1 Yu, L., Li, Z., Fang, M. & Xu, Y. Acetylation of MKL1 by PCAF regulates pro-inflammatory transcription. *Biochimica et biophysica acta. Gene regulatory mechanisms* **1860**, 839-847 (2017). https://doi.org/10.1016/j.bbagrm.2017.05.006

2 Li, Z. *et al.* Epigenetic activation of PERP transcription by MKL1 contributes to ROS-induced apoptosis in skeletal muscle cells. *Biochimica et biophysica acta. Gene regulatory mechanisms* **1861**, 905-915 (2018). https://doi.org/10.1016/j.bbagrm.2018.07.011

3 Li, Z. *et al.* The histone methyltransferase SETD1A regulates thrombomodulin transcription in vascular endothelial cells. *Biochimica et biophysica acta. Gene regulatory mechanisms* **1861**, 752-761 (2018). https://doi.org/10.1016/j.bbagrm.2018.06.004

4 Li, Z. *et al.* BRG1 regulates NOX gene transcription in endothelial cells and contributes to cardiac ischemia-reperfusion injury. *Biochimica et biophysica acta. Molecular basis of disease* **1864**, 3477-3486 (2018).
